# *Clostridium scindens* secretome suppresses virulence gene expression of *Clostridioides difficile* in a bile acid-independent manner

**DOI:** 10.1128/spectrum.03933-22

**Published:** 2023-09-26

**Authors:** Carmen Saenz, Qing Fang, Thiyagarajan Gnanasekaran, Samuel Addison Jack Trammell, June Daphne Arnold Buijink, Paola Pisano, Michael Wierer, Frédéric Moens, Bettina Lengger, Asker Brejnrod, Manimozhiyan Arumugam

**Affiliations:** 1 Novo Nordisk Foundation Center for Basic Metabolic Research, Faculty of Health and Medical Sciences, University of Copenhagen, Copenhagen, Denmark; 2 Department of Biomedical Sciences, University of Copenhagen, Copenhagen, Denmark; 3 Proteomics Research Infrastructure, Faculty of Health and Medical Sciences, University of Copenhagen, Copenhagen, Denmark; 4 ProDigest, Gent, Belgium; 5 Novo Nordisk Foundation Center for Biosustainability, Technical University of Denmark, Lyngby, Denmark; 6 Institute of Health Technology, Technical University of Denmark, Lyngby, Denmark; University of Warwick, Coventry, United Kingdom

**Keywords:** *Clostridioides difficile* infection, *Clostridium scindens*, bile acids, secretome, Human Intestinal Microbial Ecosystem, gut microbiome, omics data, pathogen, toxin suppression

## Abstract

*Clostridioides difficile* infection (CDI) is a major health concern and one of the leading causes of hospital-acquired diarrhea in many countries. *C. difficile* infection is challenging to treat as *C. difficile* is resistant to multiple antibiotics. Alternative solutions are needed as conventional treatment with broad-spectrum antibiotics often leads to recurrent CDI. Recent studies have shown that specific microbiota-based therapeutics such as bile acids (BAs) are promising approaches to treat CDI. *Clostridium scindens* encodes the bile acid-induced (*bai*) operon that carries out 7-alpha-dehydroxylation of liver-derived primary BAs to secondary BAs. This biotransformation is thought to increase the antibacterial effects of BAs on *C. difficile*. Here, we used an automated multistage fermentor to study the antibacterial actions of *C. scindens* and BAs on *C. difficile* in the presence/absence of a gut microbial community derived from healthy human donor fecal microbiota. We observed that *C. scindens* inhibited *C. difficile* growth when the medium was supplemented with primary BAs. Transcriptomic analysis indicated upregulation of *C. scindens bai* operon and suppressed expression of *C. difficile* exotoxins that mediate CDI. We also observed BA-independent antibacterial activity of the secretome from *C. scinden*s cultured overnight in a medium without supplementary primary BAs, which suppressed growth and exotoxin expression in *C. difficile* mono-culture. Further investigation of the molecular basis of our observation could lead to a more specific treatment for CDI than current approaches.

**IMPORTANCE:**

There is an urgent need for new approaches to replace the available treatment options against *Clostridioides difficile* infection (CDI). Our novel work reports a bile acid-independent reduction of *C. difficile* growth and virulence gene expression by the secretome of *Clostridium scindens*. This potential treatment combined with other antimicrobial strategies could facilitate the development of alternative therapies in anticipation of CDI and in turn reduce the risk of antimicrobial resistance.

## INTRODUCTION

Antibiotic treatments disrupt intestinal microbial communities and increase susceptibility to intestinal pathogens such as *Clostridioides difficile*, a Gram-positive and obligate anaerobic bacterium (1). *C. difficile* infection (CDI) is a major public health concern due to the rapid evolution of multidrug-resistant strains (2). It is potentially life-threatening, especially in elderly people and patients whose gut microbiota has been disrupted by broad-spectrum antibiotic treatments (1, 3). CDI is one of the leading causes of hospital-acquired diarrhea in many countries with notable mortality, morbidity, and associated healthcare costs (4, 5). Even though CDI cases can be treated effectively with antibiotics in the short term, recurrent infection relapses are frequent and problematic, affecting approximately 20–25% of patients (6, 7). Therefore, research has focused on new treatments to prevent and cure CDI.

*C. difficile* is transmitted in the form of spores, which are metabolically dormant cells resistant to harsh conditions such as the acidic environment of the stomach or the oxygenated conditions outside the host. Upon entering the gastrointestinal tract, *C. difficile* spores start to germinate in the presence of primary bile acids (BAs), resulting in vegetative cells capable of replication and pathogenesis (8). Under normal circumstances, the gut microbiota prevent the germination of these spores and subsequent *C. difficile* colonization of the colon by functioning as a barrier to the infection. This resistance could be reduced in patients with disrupted microbiota due to antibiotic treatment, which is the major risk factor for developing CDI (9 – 11).

The pathogenicity of this antibiotic-resistant bacterium is associated with the production of two homologous exotoxins, TcdA and TcdB, which are members of the large clostridial toxins family. In the host, these toxins glycosylate the Rho GTPases, disaggregate the actin cytoskeleton of the cells, promote cell death, and disrupt the intestinal epithelial barrier (12, 13). In addition, between 17% and 23% of *C. difficile*, strains produce a third toxin, the binary toxin *C. difficile* transferase, which belongs to a family of bipartite ADP-ribosylating clostridial toxins and has been associated with increased severity of CDI (12, 14). These toxins are secreted in response to several physiological and environmental signals, such as stress and nutrient limitation, as well as the detection of cell density through quorum sensing. The complex regulation of toxin genes and the conditions that trigger their expression indicate a close connection between virulence and metabolism for improving nutrient availability (15).

Primary BAs, such as cholic acid (CA) and chenodeoxycholic acid (CDCA), are synthesized from cholesterol in the liver and transported to the small intestine. A subset of low-abundance gut bacteria, such as *Clostridium scindens* (16 – 18), transforms these primary BAs into secondary BAs in humans (Fig. S1). *C. scindens* carries a bile acid-induced operon (termed *bai*) to hydroxylate CA and CDCA and produce secondary BAs deoxycholic acid (DCA) and lithocholic acid (LCA), respectively (Fig. S1). The *bai* operon contains eight genes (seven of them encoding enzymes and one encoding a transporter) (19, 20). Previous studies have reported *in vitro* inhibition of *C. difficile* growth by DCA (21, 22) or by combining it with LCA and tryptophan-derived antibiotics (23). In addition, in patients and animal models, it has been shown that secondary BAs inhibit the growth of *C. difficile* (24, 25).

Here, we studied the effect of *C. scindens* on *C. difficile* using two culture setups in an automated *in vitro* fermentor: (i) continuous co-culturing of *C. scindens* and *C. difficile* and (ii) batch culture of human donor fecal microbiota, together with *C. scindens* and *C. difficile*. We performed multi-omics analysis to investigate how the combination of *C. scindens* and BAs impacted *C. difficile* abundance and virulence gene expression in continuous and batch culture setups when primary BAs were added to the co-culture.

## RESULTS

### *C. scindens*-mediated *C. difficile* growth reduction in an automated fermentor

We used an automated anaerobic *in vitro* fermentor for both continuous and batch culture experiments to ensure consistency between experiments. Another reason to choose batch culture in the fermentor over traditional anaerobic batch culture was that the fermentor maintains physiological pH and temperature and supports complex microbial communities (26).

**Fig 1:**
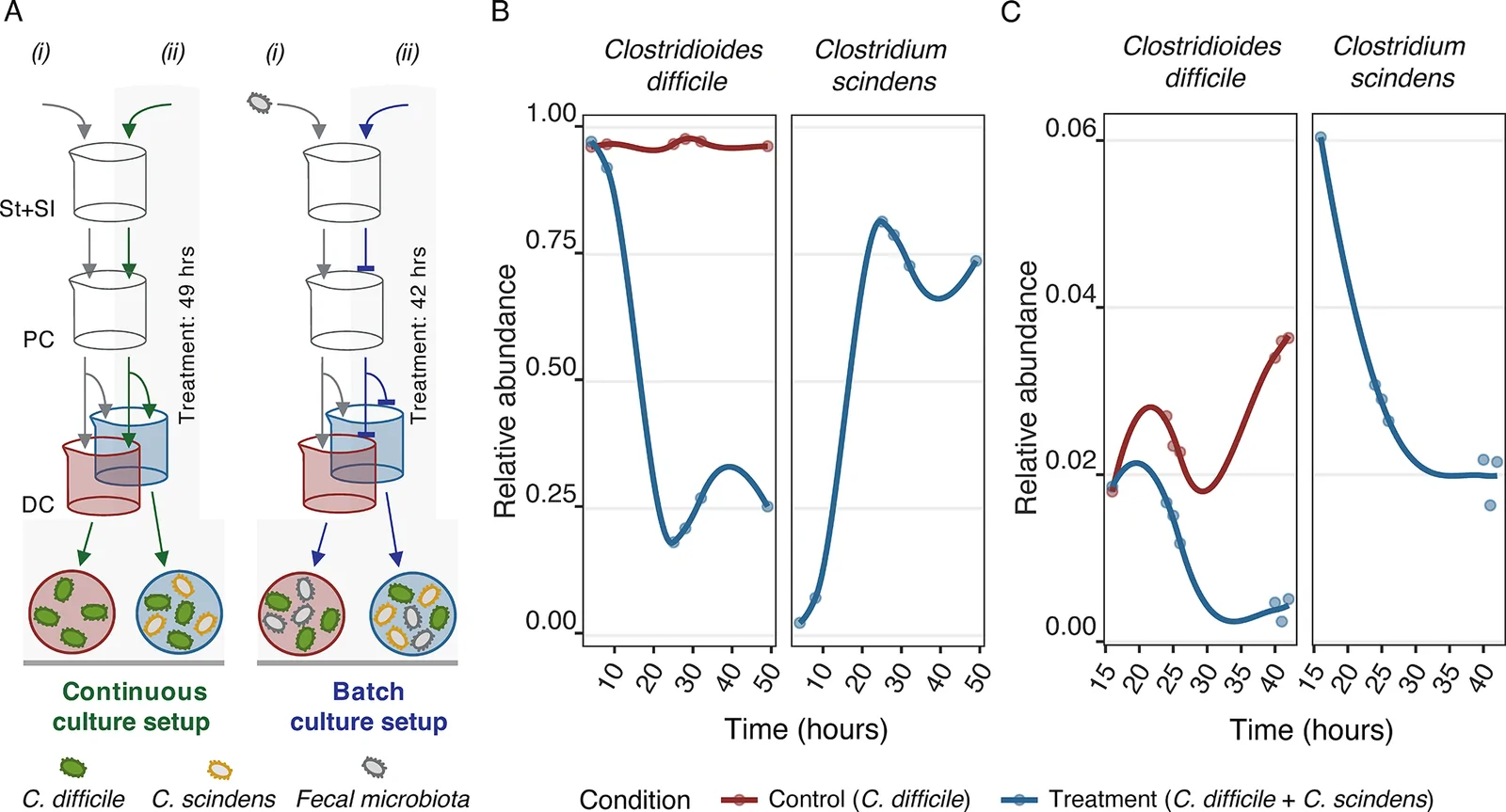
*C. scindens*-mediated *C. difficile* growth reduction in an *in vitro* fermentor with two different culture setups. (**A**) *In vitro* experiments in continuous (green) or batch (blue) culture setups. Each experiment is divided into two stages: (i) a stabilization period (left part of each diagram) in which CDI was established with the inoculation of *C. difficile* (and the fecal microbiota community) into SHIME; and (ii) a treatment period once *C. scindens* was introduced (right part of each diagram). (**B**) Relative abundance of *C. difficile* and *C. scindens* in *C. difficile* mono-culture (control) and *C. difficile + C. scindens* co-culture (treatment) conditions during the treatment period of 49 hours in a continuous culture setup. (**C**) Relative abundance of *C. difficile* and *C. scindens* when adding *C. difficile* alone (control) and *C. difficile + C. scindens* (treatment) to an existing fecal microbiota community during the treatment period of 42 hours in a batch culture setup. Local regression fitting was used to connect the points in panels **B** and **C** (see Materials and Methods).

First, we cultured *C. difficile* alone (control condition) or *C. difficile* with *C. scindens* (treatment condition) for 49 hours (Fig. 1A). Using 16S rRNA gene V4 variable region amplicon data, we quantified the relative abundance of these two bacteria (see Materials and Methods). In the control condition, *C. difficile* levels were stable throughout the experiment (median relative abundance 0.97). On the contrary, its relative abundance decreased between 8 and 25 hours of co-culture with *C. scindens* in the treatment condition (Fig. 1B; Fig. S2A). The longitudinal trends of the beta diversity captured by the first two coordinates of a principal coordinate analysis (PCoA) performed using Bray-Curtis dissimilarity showed no differences between the control and treatment conditions during the first 8 hours (see Materials and Methods; Fig. S2B). However, after 25 hours, a strong separation between samples from control and treatment conditions was observed in the first principal coordinate that explained 99.6% of the variance. This separation could be explained by the decreased relative abundance of *C. difficile* in the co-culture at the corresponding timepoints (Fig. 1B). As relative abundance data are compositional in nature, we next investigated whether *C. difficile* was reduced in absolute abundance. We observed a reduction in *C. difficile* colony-forming units (CFU) in the treatment condition compared with the control condition (Fig. S3). We also note that three additional ASVs were identified, corresponding to *Ralstonia*, *Pseudolabrys*, and an unknown ASV from the Burkholderiales class. These three ASVs were detected at median relative abundance rates of 3% and 0.3% in the control and treatment samples, respectively (Fig. S2A). These contaminating microbial DNA could have been introduced during sample preparation. *Ralstonia* has been reported in the literature as a water- and soil-associated bacterial genus (27), while *Pseudolabrys* has been identified as a normal member in soil and dust samples (28, 29). Nevertheless, their relative abundance is low, suggesting low overall contamination.

Under biological conditions, bacteria compete for nutrients and interact with neighboring microbial species. This complexity was not captured in the culture setup used above. To overcome this shortcoming, we then used a second culture setup to introduce *C. difficile* and *C. scindens* into a feces-derived microbiota community maintained in the *in vitro* fermentor (Fig. 1A). In this experiment, the establishment of the fecal microbial community, its dysbiosis, and *C. difficile* colonization phases were run for 8 weeks under controlled pH conditions with a standard flow of medium until *C. scindens* was inoculated (treatment phase). During the treatment phase, there was no flow of medium, simulating a batch fermentor with diminishing nutrient availability (batch culture setup; see Materials and Methods). The samples were collected at 16, 24, 25, 26, 40, 41, and 42 hours for both control and treatment conditions. We profiled shotgun metagenomic high-quality reads from these 14 samples at the species level, identifying 55 taxa (see Materials and Methods). As seen in Fig. S2C, the overall bacterial genus composition between the two conditions differed. In the presence of *C. scindens*, *Coprococcus* had a higher relative abundance (median 9.1 × 10^−2^ vs 3.4 × 10^−5^), while *Hungatella*, *Eggerthella*, and *Eisenbergiella* had a lower relative abundance (median 1.9 × 10^−4^ vs 7.4 × 10^−3^, 1.6 × 10^−4^ vs 2.2 × 10^−3^, and 1.1 × 10^−4^ vs 1.2 × 10^−3^, respectively). Interestingly, the relative abundance of *C. difficile* increased twofold in the control condition, while it decreased 3.6-fold in the presence of *C. scindens* (Fig. 1C). In addition, we observed a threefold decrease in the relative abundance of *C. scindens* reaching a stable level after 26 hours in the treatment condition. Similar to our observations in Fig. S2B, a PCoA on the taxonomic relative abundance profiles showed that the two conditions were more similar at the earlier timepoints but started to diverge from each other over time (Fig. S2D). When investigating the absolute abundance of *C. difficile*, we observed a significant decrease in the treatment condition compared with the control condition when quantifying the total number of cells via qPCR detection (Student’s *t*-test, Fig. S4).

### Changes in the expression of the 7α-dehydroxylation pathway in *C. scindens*

**Fig 2:**
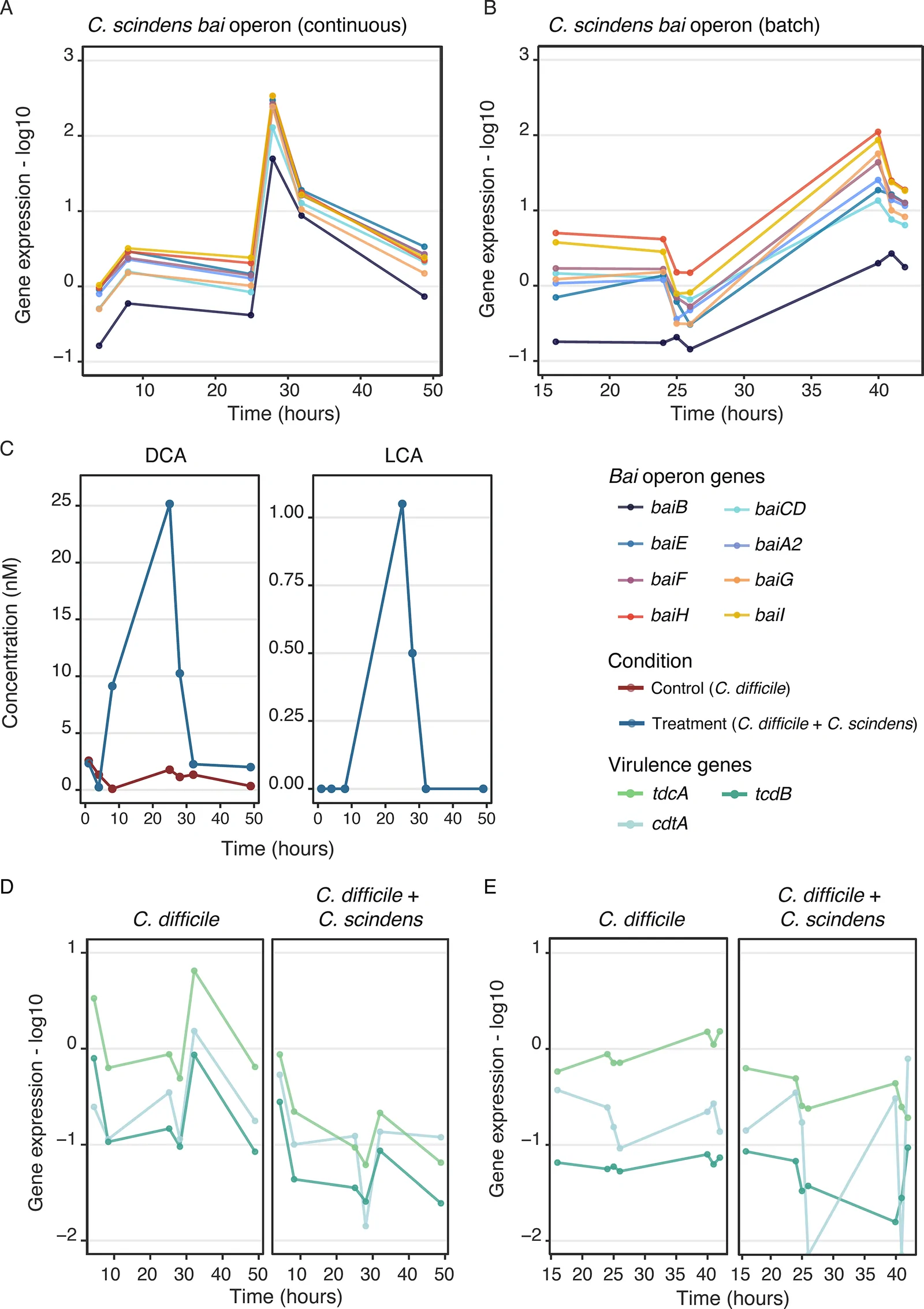
*C. scindens*-mediated biotransformation of primary BAs and downregulation of *C. difficile* toxin tcdA expression. (**A and B**) Gene expression (log 10 transformed) of the *bai* operon in *C. scindens* in (**A**) continuous culture or (**B**) batch culture setups. (**C**) Concentration of DCA and LCA in the treatment condition compared with the control condition in a continuous culture setup using targeted metabolomics. (**D and E**) Gene expression (log 10 transformed) of the virulence genes *tcdA*, *tcdB*, and *cdtA* in *C. difficile* in (**D**) continuous culture or (**E**) batch culture setups.

We next investigated the mechanisms behind the reduction of *C. difficile* relative abundance in the presence of *C. scindens*. Colonic bacterium *C. scindens* carries the *bai* operon, which biotransforms primary BAs CA and CDCA into secondary BAs DCA and LCA, respectively (Fig. S1). Both DCA and LCA are known to inhibit *C. difficile* growth (21, 30, 31). To investigate differential gene expression in *C. scindens* between conditions, we sequenced the total mRNA present in the samples from both culture setups. We used metatranscriptomic data to compute gene expression profiles that were not confounded by species abundances after adjusting mRNA abundances by the transcripts of 10 universal single-copy phylogenetic marker genes (MGs) from the corresponding species (see Materials and Methods). We then specifically visualized the expression of the eight genes encoded in the *C. scindens bai* operon in both continuous and batch culture setups when this bacterium was present in the media. In the continuous culture setup, the expression was especially increased between 82.3- and 161-fold after 28 hours (Fig. 2A), whereas in the batch culture setup, the gene expression was notably increased between 9.3- and 46.7-fold after 40 hours. The highest expressed gene was *baiH*, encoding an NADH:flavin oxidoreductase (Fig. 2B). After 41 hours, there was a decrease in the gene expression. To investigate the effects of this upregulation of *bai* operon, we performed targeted metabolomics in the continuous culture setup, where the fecal microbial community was not present (see Materials and Methods). In total, 26 BA standards were used for quantification, among which we detected 20 BAs across all the samples (Table S1). Between hours 8 and 32, we observed an increase in the concentration of secondary BAs DCA and LCA compared with the control condition (Fig. 2C). This suggests that the increase in expression of the *bai* operon genes observed during the same period (Fig. 2A) resulted in increased production of secondary BAs.

### Reduction of *C. difficile* toxin expression in the presence of *C. scindens*

The pathogenicity of *C. difficile* is associated with the expression of *tcdA* and *tcdB* genes that encode the toxins TcdA and TcdB, respectively, which are primarily responsible for the symptoms associated with CDI (32, 33). In the continuous culture setup, the expression of the genes *tcdA*, *tcdB*, and *cdtA* was reduced in the presence of *C. scindens* (Fig. 2D). The highest reduction was observed in *tcdA* gene expression with 8.2× reduction in median expression compared with the control (4.2× for *tcdB* and 2.5× for *cdtA*). In addition, in the treatment condition, this gene was expressed almost four times less after 49 hours. On the other hand, when we inspected the expression of all three virulence genes during 42 hours in the batch culture setup, we observed a reduction in the expression of *tcdA,* but we did not see a clear trend of downregulation of these toxin genes (Fig. 2E).

### *C. scindens* overnight spent medium affects *C. difficile* toxin expression in a bile acid-independent manner

**Fig 3:**
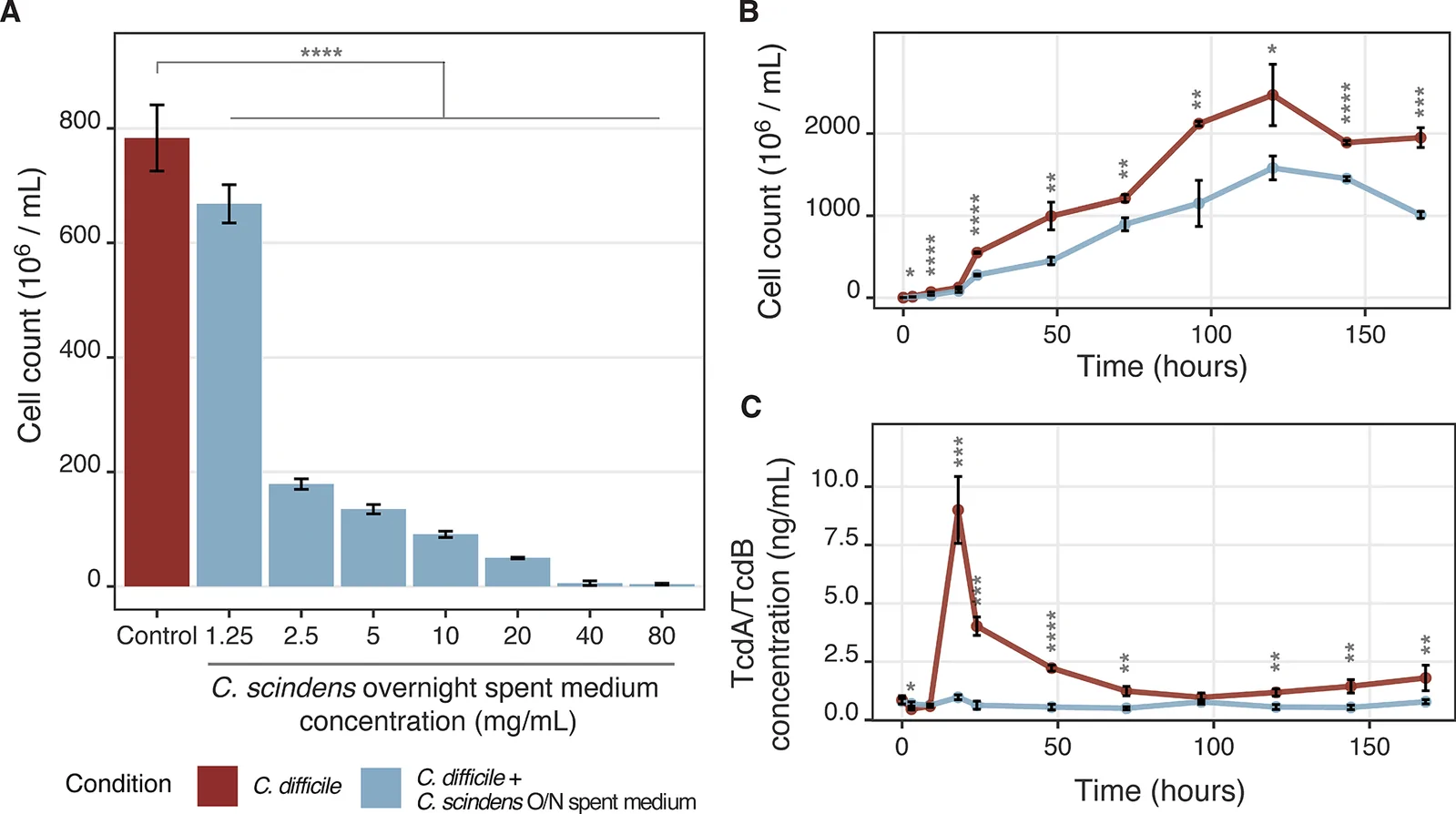
Bile acid-independent effect of CsOSM in TcdA/TcdB toxin expression and growth of *C. difficile*. (**A**) Cell counts from *C. difficile* batch cultures treated with different concentrations of freeze-dried CsOSM for 40 hours. Unpaired two-sided Student’s *t*-tests were performed to compare the *C. difficile* cell counts between each concentration of CsOSM and that of the control group. (**B**) Cell counts from *C. difficile* batch cultures treated with 5 mg/mL CsOSM during 7 days post-treatment. (**C**) TcdA/B toxin concentration in *C. difficile* batch cultures measured using ELISA. All the *P*-values have been adjusted by false discovery rate (FDR) (* adjusted *P* < 0.05, ** adjusted *P* < 0.01, *** adjusted *P* < 0.001, and **** adjusted *P* < 0.0001. *n* = 3 independent experiments).

Our results so far suggested that *C. scindens* reduced the relative abundance of *C. difficile* and the expression of toxin genes *tcdA* and *tcdB*. However, it was unclear whether live *C. scindens* was needed or whether its secreted molecules could have had the same effect. To investigate this further, we decided to treat *C. difficile* cultures with *C. scindens* overnight spent medium. To choose an optimal culture medium, we cultured *C. difficile* in two different media (standard nutritional medium and brain heart infusion [BHI] growth medium) using culture flasks and quantified the expression of TcdA and TcdB toxins by proteomics (see Materials and Methods). As seen in Fig. S5, TcdA toxin levels were higher when *C. difficile* was cultured in BHI medium compared with standard nutritional medium, and TcdB was not detected in the latter. Thus, we chose BHI medium to culture *C. difficile* for the following experiments (see Materials and Methods). Then, to choose an optimal dose of CsOSM, we carried out a dose-response assay by adding different amounts of freeze-dried CsOSM on *C. difficile* mono-culture (see Materials and Methods). Addition of CsOSM did not alter pH, which remained unchanged at 6.7 ± 0.05 (*n* = 3; Table S2). When cultivating *C. difficile* in the presence of CsOSM for 40 hours, cell counts reduced dramatically (over a threefold reduction) with a concentration of 2.5 mg/mL or above (Fig. 3A). Based on these results, we chose 5 mg/mL as the optimal dose for further investigation.

We cultivated *C. difficile* overnight in BHI medium, diluted it to 10^6^ cells/mL next day, and immediately added freeze-dried CsOSM (T0). We continued the culture and sampled at 11 timepoints over 7 days (0, 3, 9, 18, 24, 48, 72, 96, 120, 144, and 168 hours). For each specific sampling timepoint, we recorded the cell count and quantified TcdA and TcdB toxins using enzyme-linked immunosorbent assay (ELISA) (see Materials and Methods). CsOSM treatment caused a significant reduction in *C. difficile* cell count throughout the experiment (Fig. 3B; adjusted *P* < 0.05 in 9 out of 10 timepoints, Student’s *t*-test; between 1.3- and 2.2-fold reduction). It also caused a significant suppression of TcdA/TcdB toxins (Fig. 3C; adjusted *P* < 0.001 from 18 to 48 hours, Student’s *t*-test). While the toxins reached a peak concentration of 8.3 ng/mL at 18 hours in the control condition (13.5-fold increase from 9 hours), they remained low at 0.9 ng/mL after CsOSM treatment (1.5-fold increase from 9 hours) suggesting suppression of toxin production by CsOSM.

It is important to note that, contrary to the continuous and batch culture setups, no primary BAs were added to the BHI medium used to grow *C. scindens* to harvest CsOSM. However, when performing targeted metabolomics for 26 BAs in BHI medium and CsOSM, we detected 11 of them in at least two of the three biological replicates (Table S3; see Materials and Methods), including CA, CDCA, and DCA (Fig. S6A). The concentration of DCA in the BHI medium was 10^5^ times lower compared with that of the standard feed used in continuous and batch culture setups. In the literature, it has been reported that 50 µM of CA had no significant effect on the growth of *C. difficile* and 50 µM DCA inhibited (~25%) *C. difficile* (23). Since such information was missing specifically for DCA in BHI medium and at lower concentrations, we performed a dose-response assay using different concentrations of DCA on a *C. difficile* mono-culture (see Materials and Methods). Addition of 5 µM DCA had negligible effects to the growth curve, while the difference was considerable at 10 µM and above (Fig. S6B). This suggests that the presence of DCA in the growth medium and CsOSM at femtomolar (1e^−9^ μM) concentrations (Fig. S6A) could not explain the inhibitory effects of CsOSM. Thus, we conclude that CsOSM leads to reduction of both *C. difficile* cell growth and TcdA/B toxin levels whether primary BAs were added to the growth media or not. Several mechanisms could cause our observed results, such as sporulation inhibition or direct inhibition of some regulatory processes by metabolites (23, 34). Comparing cell morphology using transmission electron microscopy (TEM), CsOSM-treated *C. difficile* mono-culture had a significantly lower fraction of normal vegetative cells and a significantly higher fraction of spores than control culture (Student’s *t*-test, adjusted *P* < 0.0001), suggesting that CsOSM induces the sporulation of *C. difficile* cells (Fig. S7). CsOSM had this effect even though it contained CA (Fig. S6A), which is known to be a germinant for *C. difficile* spores. This could be due to the extremely low amounts of CA (femtomolar concentration) in CsOSM, whereas studies have reported that 1–5 mM CA is required to work as a germinant for *C. difficile* spores (21, 30, 31, 35).

## DISCUSSION

*C. difficile* infection is a critical healthcare problem, and its treatment with broad-spectrum antibiotics leads to gut microbiota disruption (1, 24, 36 – 39). The gut commensal *C. scindens* was shown to be associated with resistance to CDI in animal models and human patients, potentially mediated by the synthesis of secondary BAs, specifically DCA, via its *bai* operon (23, 24, 40, 41). Further studies reported *C. difficile* abundance reduction when co-cultured with *C. scindens* (21, 22).

Here, we observed similar results when we co-cultured *C. difficile* and *C. scindens* in a primary BA-containing medium in a continuous culture setup. *C. difficile* relative abundance as well as absolute abundance measured by CFU was reduced after 49 hours of co-culture (Fig. 1B; Fig. S3). Even though we identified three additional (contaminating) microbial strains, their low abundance (<3.8% combined relative abundance) suggests a limited impact of contamination on our results (Fig. S2A). When we added *C. difficile* alone or together with *C. scindens* to an existing fecal microbiota culture, we also observed a significant reduction in relative abundance of the total number of cells of *C. difficile* in the presence of *C. scindens* (Fig. 1C; Fig. S4).

A previous study reported the temporal change in the expression of *C. difficile* toxin genes *tcdA* and *tcdB* during 48 hours of batch mono-culture, with the toxin gene expression starting at 12 hours, peaking at 24 hours in the stationary phase and gradually decreasing in the next 24 hours (42). In our batch mono-culture experiments, the production of *C. difficile* TcdA/TcdB toxins reached the highest level at 18 hours and was in a downward trend during the next 150 hours (Fig. 3C). In contrast, we observed a sustained gene expression of *tcdA*, *tcdB*, and *cdtA* in *C. difficile* mono-culture when using an *in vitro* fermentor that continuously supplied fresh medium. However, when *C. difficile* was co-cultured with *C. scindens*, we detected a reduction in gene expression of these toxins. At the same time, *C. scindens* exhibited increased expression of all eight genes in the *bai* operon, reaching their peak expression at the same time as peak concentration of DCA. Given that the medium was supplemented with pancreatic juice containing primary BAs, our results agree with a previous hypothesis that biosynthesis of DCA by *C. scindens bai* operon genes could be behind *C. difficile* inhibition (23, 24).

However, the suppression of *C. difficile* growth can be caused in a BA-independent manner (20) such as by the competition of nutrients that are important for the pathogen colonization (34, 43, 44). We went further and investigated whether the secretome of *C. scindens* cultured without specific supplementation of primary BAs could have had the same effect. Indeed, it decreased both *C. difficile* absolute abundance and TcdA/B toxin levels. Furthermore, TEM images showed that this secretome led to a significantly higher proportion of spores and a significantly lower proportion of normal vegetative cells (Fig. S7B, Student’s *t*-test, adjusted *P* < 0.0001). These results suggest a BA-independent effect of *C. scindens* on *C. difficile* abundance and production of TcdA/B toxins in CDI.

It has been shown that the effect on *C. difficile* growth in the presence of *C. scindens* is dependent on the strain of the pathogen (20). Therefore, we suggest that a deeper understanding of the mechanisms and the roles of TcdA/B in *C. difficile* virulence and targeting their gene expression without using a probiotic could be more attractive for drug development. There are already such solutions, most recently reported alternatives being auranofin, which decreases toxin and sporulation production in infected mice and in *in vitro* setups (45); ebselen, which kills *C. difficile* by disrupting its redox homeostasis (46); and HMOs, which have been reported as having the potential to combat CDI recurrence, but their exact antipathogenic mechanisms of action have not been elucidated (47). Given that we have observed the BA-independent effects of *C. scindens*, further work on identification and validation of the bioactive molecule(s) combined with other antimicrobial strategies (48) could lead to novel alternative treatments for CDI.

## MATERIALS AND METHODS

### *C. difficile* and *C. scindens in vitro* cultivation setup designs and sample collection

*C. difficile* ([LMG 21717 [ribotype 1]) and *C. scindens* (DSM 5676) were procured from Belgian coordinated collection of microorganisms (Ghent, Belgium) and German collection of microorganisms and cell culture GmBH (Braunschweig, Germany). *C. difficile* was grown alone and in the presence of *C. scindens*, as control and treatment conditions, in the Simulator of the Human Intestinal Microbial Ecosystem (SHIME) (49). The system was used in two different setups: continuous and batch culture setups. All SHIME culture experiments were done at Prodigest BVBA, Ghent, Belgium.

For the continuous culture setup, we specifically used the luminal SHIME (Prodigest BVBA, Ghent, Belgium) with the following configuration: a combined stomach and small intestine (ST + SI) compartment, a proximal colon (PC) compartment, and two distal colon (DC) compartments connected to the same PC (Fig. 1A). Throughout the experiment, all the compartments were maintained at a constant temperature of 37°C and continuously stirred at 300 rpm. All the compartments were tightly sealed and flushed with 100% N_2_ to maintain an anaerobic environment. The volume and the pH of the colon compartments were maintained as follows: PC (pH = 5.6–5.9, *V* = 500 mL) and DC (pH = 6.6–6.9; *V* = 800 mL). The ST + SI compartment is a sterile compartment that operates on a fill-and-draw principle, while the colon compartments are fed-batch reactors inoculated with bacteria of interest. During every feeding cycle (8-hour equal interval), the ST + SI compartment is supplied with sterile standard nutritional medium (SF, 140 mL) (50) and pancreatic juice (60 mL) containing bile acids and pancreatic enzymes. The resulting nutritional medium mixture is incubated for 1 hour simulating *in vivo* digestion. Thereafter, the digested nutritional media from ST + SI compartment are transferred to the PC compartment, and simultaneously, the contents from the PC are transferred to the DC and the excess content from DC to waste at a specific flow rate of 3.5 mL/minute. In addition to SF, an additional amount (20% of nutrients) of a simple sugar mix consisting of glucose, fructose, galactose, and lactose was directly added to the PC during every feeding cycle with an assumption that 25% of these nutrients will reach DC. This addition of simple sugars was performed since the metabolic potential of the *C. scindens* type strain (JCM6567 ~identical to DSM 5676) to degrade carbohydrates was mostly specialized towards the degradation of simple sugars such as glucose, ribose, mannose, fructose, galactose, and lactose, while the strain was unable to ferment polysaccharides such as starch (51). The pancreatic juice contained NaHCO_3_ (12.5 g/L), oxgall (6 g/L), and pancreatin (0.9 g/L) with 25% of additional CDCA (25% of CA concentration). This is because bovine BAs (oxgall) are known to contain higher amounts of CA compared with CDCA (52). Both DC compartments were inoculated with *C. difficile* overnight, followed by the introduction of *C. scindens* in one of the DC compartments as treatment. The treatment was performed for 49 hours from the time *C. scindens* was added. The sampling was performed at 4, 8, 25, 28, 32, and 49 hours during the treatment phase, and the collected samples were stored at −80°C until further use.

For the batch culture setup, initially both the PC and DC compartments were inoculated with a microbial community derived from a human fecal sample obtained from an anonymous donor. Then, to induce the infection of *C. difficile* in DC, dysbiosis of the established community using clindamycin was performed for a period of 5 days with simultaneous inoculation of *C. difficile*. The culture setup was monitored for a period of 5 weeks until a stable colonization of *C. difficile* was observed. During these 5 weeks, the feeding cycles and transfer of liquids were performed similar to the continuous culture setup. Then, as a treatment, *C. scindens* was introduced into the *C. difficile*-infected DC compartment. During the treatment phase, the PC and DC compartments were switched to the batch mode by stopping the feeding cycles and transfer of liquids between the compartments. However, the respective pH levels of both PC and DC were strictly maintained during the entire treatment phase. The treatment was performed for 42 hours from the time *C. scindens* was added. The samples were collected during the treatment phase at 16, 24, 25, 26, 40, 41, and 42 hours and stored at −80°C until further use.

### *C. difficile* CFU measurement

Tenfold dilution series (1/10^2^ to 1/10^5^ dilutions for each sample) were prepared from the collected samples in anaerobic phosphate-buffered saline, and subsequently, 0.1 mL was transferred to pre-reduced petri dishes containing the commercial BD Clostridium Difficile Agar with 7% Sheep Blood (Becton, Dickinson and Company). Plates were incubated anaerobically for 72 hours before counting.

The total viable counts (TVC) of bacteria in a sample are expressed in CFU per mL, using the formula $N=\frac{C*d}{V}$ , where *C* is the number of counted colonies, *d* is the dilution, i.e., the reciprocal of the dilution factor, and *V* is the volume that was brought on the agar plate (in mL, in this case 0.1 mL). Since we used five dilutions in the tenfold dilution series, we calculated TVC using the formula $N=\frac{\Sigma C}{\Sigma \frac{V}{d}}$ .

### *C. difficile* qPCR

*C. difficile*-selective qPCR was performed using the protocol described by Matsuda et al. (53). The qPCR protocol targeted the 23S rRNA gene, using primer sets Cd-lsu-F (5′-GGG AGC TTC CCA TAC GGG TTG-3′) and Cd-lsu-R (5′-TTG ACT GCC TCA ATG CTT GGG C-3′). The amplification program consisted of one cycle at 95°C for 15 minutes, followed by 40 cycles at 94°C for 20 s, 60°C for 20 s, and 72°C for 50 s. Amplification and detection were performed using the QuantStudio 5 Real-Time PCR System (Applied Biosystems).

### DNA and RNA extraction

The DNA extractions from the samples were performed using DNeasy Ultraclean microbial kits (Qiagen, Germany) according to the manufacturer’s protocol. The quality of DNA and its concentrations were assessed using a NanoDrop system and a Qubit Fluorometer/microplate reader (Thermo Fisher Scientific, USA).

The RNA extractions from the samples were performed using the RNeasy mini kit (Qiagen, Germany) according to the manufacturer’s protocol. Ribosomal RNA was removed from the total RNA using the Illumina Ribo-Zero rRNA kit (Illumina, USA) according to the manufacturer’s protocol.

### 16S rRNA gene amplicon and metatranscriptomic sequencing of samples from the continuous culture setup

The 16S ribosomal RNA gene amplicon sequencing targeting the V4 variable region was performed using Illumina Hiseq 2500 at BGI Europe (Copenhagen, Denmark), generating 2 × 250-bp paired-end reads. A total of 499,598 16S rRNA gene raw paired-end reads (mean 41,633 read pairs per sample, ranging from 41,157 to 42,040) were obtained from 12 *in vitro* samples in the continuous culture setup.

Metatranscriptomic sequencing was performed using BGISEQ (2 × 150-bp paired-end sequencing) at BGI Europe (Copenhagen, Denmark). A total of 263.25 million raw paired-end metatranscriptomic reads (mean 21.94 million read pairs per sample, ranging from 20 to 24.81 million) were produced from 12 *in vitro* samples that matched the 16S rRNA ones.

### Shotgun metagenomic and metatranscriptomic sequencing of samples from batch culture setup

Shotgun metagenomic sequencing and metatranscriptomic sequencing were performed using BGISEQ (2 × 150-bp paired-end sequencing) at BGI Europe (Copenhagen, Denmark). A total of 513.62 million shotgun metagenomic raw read pairs (mean 36.69 million read pairs per sample, ranging from 28.84 to 43.14 million) and 333.13 million raw paired-end metatranscriptomic reads (mean 23.80 million read pairs per sample, ranging from 20.72 to 25.73 million) were obtained from 14 *in vitro* paired samples.

### 16S rRNA gene amplicon sequence pre-processing

We used the DADA2 v1.18 R package (54) to process the 2 × 250-bp Hiseq 2500 PE250 Dual-Index amplicon sequencing reads representing the V4 region of 16S rRNA genes. Primer-end reads were removed using the following parameters: “trimLeft = 10, truncLen = 0, truncQ = 2, maxN = 0, maxEE = 0.5, minOverlap = 20, maxMismatch = 0, pool = FALSE.” The error-rate-learning step was performed with the parameter nreadsLearn = 1.2e + 06. We obtained 365,155 high-quality paired-end reads (mean 30,430 read pairs, ranging from 28,430 to 32,589). This resulted in an amplicon sequence variant (ASV) table. Chimeras were identified and removed from the amplicons, resulting in a table of 19 unique ASVs. A prevalence filter was applied on the presence of these ASV in at least three samples, reducing the set of ASVs to five.

### Taxonomic classification of ASV sequences from 16S rRNA gene amplicon sequences

Following the recommended procedure from DADA2 developers, taxonomic classification of ASV sequences was performed using the SILVA database (55), version 138. To assign the taxonomy up to the genus level, we used the assignTaxonomy function (with silva_nr_v138_train_set.fa), followed by the addSpecies function (with silva_species_assignment_v138.fa) to assign species using exact sequence matching. Both functions are from DADA2 (54).

### Alpha- and beta-diversity analyses

Alpha- and beta-diversity analyses were performed using the phyloseq package (v1.34.0). The alpha-diversity measures were determined based on rarefied data at 2,000 reads/sample. Beta diversity analysis was done by calculating the Bray-Curtis distance (56). The effects of diverse components on beta-diversity analysis were calculated using the Adonis permutational multivariate analysis of variance test from the package vegan (v2.5–7) (57).

### Metagenomic and metatranscriptomic pre-processing

Shotgun metagenomic and metatranscriptomic data were pre-processed independently of each other using the MIntO pipeline (58), which filters the raw reads by quality and read length, host genome, and rRNA sequences. Trimmomatic v0.39 (59) was used twice in the pre-procesing. First, it was utilized to remove low quality bases and sequencing adapters, which were provided by BGI (TRAILING:5 LEADING:5 SLIDINGWINDOW:4:20 ILLUMINACLIP:{adapters.fa}:2:30:10). Following this, reads that are too short were removed by setting the MINLEN parameter in Trimmomatic to 50 bp, which was estimated as the maximum read length over which 95% of the reads are kept (by setting perc_remaining_reads: 95 in MIntO). Host-derived sequences were removed by aligning the read pairs to the human genome (build hg38) using the BWA aligner (60) parameters: “bwa-mem2 mem -a.” The aligned read pairs were identified and excluded from the FASTQ files by msamtools v1.0.3 (61) (filter -S -l 30) and mseqtools (available at https://github.com/arumugamlab/mseqtools) version 0.9.1 (subset --exclude --paired --list {listfile}).

Even if the rRNA sequences were depleted prior to sequencing the RNA samples, MIntO checks for rRNA sequences in the metatranscriptomic reads. SortMeRNA v4.3.4 (62) and a locally installed database were used (“--paired_in --fastx --blast 1 --sam --other --ref”). The database comprises eukaryotic (18S and 28S) and prokaryotic (16S and 23S) rRNA sequences (available at https://github.com/biocore/sortmerna/tree/master/data/rRNA_databases).

In the batch setup, we obtained 459.53 million shotgun metagenomic high-quality host-free paired-end reads (mean 32.82 million read pairs, ranging from 25.55 to 38.84 million).

We obtained a total of 231.93 million metatranscriptomic high-quality host- and rRNA-free paired-end reads (mean 19.33 million read pairs, ranging from 17.36 to 22.32 million) and a total of 281.24 million metatranscriptomic high-quality host- and rRNA-free paired-end reads (mean 20.09 million read pairs, ranging from 15.73 to 22.69 million) from the continuous and the batch setups, respectively.

### Taxonomic profiling of shotgun metagenomes

Shotgun metagenomic taxonomic profiling was performed using the MIntO pipeline (58). High-quality filtered reads were profiled by the default program, MetaPhlAn3 v3.0.13 (63). Principal component analysis was done by calculating the Bray-Curtis distance (56) and the R package vegan (v2.5–7) (57).

### Gene expression computation

Metagenomic and metatranscriptomic high-quality filtered reads were aligned to the reference genomes (*C. difficile* LMG 21717 [ribotype 1] and *C. scindens* [DSM 5676]) followed by transcripts per million (TPM) normalization. This step was computed using the MIntO pipeline (58).

The gene expression profiles were calculated by normalizing the TPM-normalized transcript abundances by the median abundance of 10 universal single-copy phylogenetic MGs from the corresponding species, following an approach similar to Salazar et al.’s study, but customized to each individual genome (64). These MGs are constitutively expressed housekeeping genes across many different conditions (64 – 66) and are identified in each genome by FetchMGs v1.2 (available at http://motu-tool.org/fetchMG.html) as OGs: COG0012, COG0016, COG0018, COG0172, COG0215, COG0495, COG0525, COG0533, COG0541, and COG0552.

### Targeted bile acid measurement in culture supernatants and non-cultured media

Culture supernatants were removed from −20°C storage and diluted 1/100th in liquid chromatography mass spectrometry (LC-MS) grade methanol. Non-cultured BD Difco Bacto Brain Heart Infusion media, referred hereafter as media samples, were prepared fresh on the day of analysis and placed on wet ice. Twenty microliters of media samples was combined with 80 µL of LC-MS methanol and vortexed briefly (~3 s, maximum setting). Medium extracts were clarified with centrifugation at 14,000 × *g* for 10 minutes. Culture supernatants were vortexed (~3 s, maximum setting) and centrifuged for 10 minutes at 20,238 × *g*. Ten microliters of diluted culture supernatant was further diluted (1/100) in LC-MS grade water. Five microliters of diluted culture supernatant or 100 µL of methanolic media was combined with 1.88 µL of 0.1 ppm of deuterated internal standards (TLCA-d4, TDCA-d4, DCA-d4, CA-d4, TCA-d4, TDCA-d4, LCA-d5, GCA-d4, GUDCA-d4, GCDCA-d4, GDCA-d4, and CDCA-d4) for the analysis of CDCA, GCA, GCDCA, GDCA, GLCA, GUDCA, HDCA, LCA, MCA gamma, muricholic acid, TCA, TCDCA, TDCA, THDCA, TLCA, TMCA alpha, TMCA beta, TMCA gamma, TMCA omega, TUDCA, and UDCA. A standard curve was composed by combining 5 µL of standard (5 × 10^−4^, 1 × 10^−3^, 5 × 10^−3^, 0.01, 0.05, 0.1 ppm of CA, CDCA, DCA, GCA, GCDCA, GDCA, GLCA, GUDCA, hyodeoxycholic acid [HDCA], LCA, MCA alpha, MCA beta, MCA gamma, MCA omega, muricholic acid, TCA, TCDCA, TDCA, tauro-HDCA [THDCA], TMCA alpha, TMCA beta, TMCA gamma, TMCA omega, TLCA, TUDCA, and UDCA) with 1.88 µL of 0.1 ppm of deuterated internal standards (see above). An internal standard blank containing LC-MS grade water instead of standard was also composed in the same manner. Standard quality controls (QCs) were composed in duplicate by adding 2 µL of 0.1 ppm external standard. Two blanks were composed by replacing the sample volume with water and excluding internal standard. Samples, standards, and QCs were dried via speed vacuum for approximately 1 hour. The dried samples were then suspended in 50 µL of LC-MS grade water/LC-MS methanol (4:1). Ten µL of each cultured supernatant sample was combined in one vial to form a QC-pooled sample. Samples were placed in a pre-chilled autosampler held at 8°C in a random order. Ten µL of each sample was injected. The QC pool and QC standards were injected at the beginning, middle, and end of each queue. Analytes were separated over a Waters BEH C18 column (100 mm length × 2.1 mm internal diameter and 1.7 µm particle size) heated to 50°C using a gradient at a flow rate of 0.4 mL/minute. Mobile phase A and mobile phase B were composed of 0.01% formic acid (LC-MS grade) in water (LC-MS grade) and 0.01% formic acid (LC-MS grade) in acetonitrile (LC-MS grade), respectively. The gradient used is included in Table S4.

Electrospray-produced ions were detected in a negative, multiple reaction monitoring (MRM) mode on a Waters Xevo TQ-XS triple quadrupole mass spectrometer. The MRM transitions are displayed in Table S5. BAs were quantified using the internal standard normalized curve. The retention times and internal standard used for quantitation are displayed in Table S6. The following parameters were used for the analysis: capillary voltage (kV) = 2.4, cone voltage (V) = 35, source temperature (°C) = 150, desolvation temperature (°C) = 650, cone gas flow (L/hour) = 150, desolvation gas flow (L/hour) = 1,000, collision gas flow (mL/minute) = 0.14, and nebulizer gas flow (bar) = 7. The limit of quantification was estimated based on the lowest concentration included in the standard curve. Any bile acid absent in 50% or more of the samples was removed.

### *C. difficile* batch mono-culture supplemented with *C. scindens* overnight spent medium (CsOSM) or DCA

*C. difficile* and *C. scindens* were revived in BHI medium (Fisher Scientific, DK) from their respective cryostocks under anaerobic conditions (85% N2, 10% CO2, and 5% H2) in an anaerobic chamber (Coy laboratories, USA) until the stationary phase was reached. In order to create CsOSM, the overnight culture (250 µL) of *C. scindens* was passaged into a 5-mL BHI medium and grown until the stationary phase. It was then diluted in the same fresh medium to obtain a final inoculum of 10^6^ cells/mL. The diluted culture was then incubated overnight at 37°C, 250 RPM in a benchtop orbital shaker (MaxQTM 4450; Thermo Fisher Scientific, USA). On the next day, the spent medium was carefully harvested by centrifugation and subjected to filter sterilization using a 0.22-µm filter (Corning Costar Spin-X, Sigma, DK) followed by freeze drying (CoolSafe Freeze Dryers, LaboGene, DK) for 17 hours. The lyophilized CsOSM powder was stored at 4°C until use.

For the dose response assay of CsOSM, *C. difficile* preculture (5% [vol/vol]) was prepared in a 5-mL BHI medium and grown until the stationary phase using the benchtop orbital shaker at 37°C, 250 RPM in the anaerobic chamber. It was then diluted in the fresh BHI medium to obtain a final inoculum of 10^6^ cells/mL in order to be used for the treatment with CsOSM. Lyophilized CsOSM powder was weighed and dissolved in *C. difficile* culture in 1.25, 2.5, 5, 10, 20, 40, and 80 mg/mL at T0 of a 40-hour time course cultivation experiment.

For the time course study to investigate the effect of CsOSM, *C. difficile* preculture (5% [vol/vol]) was prepared in a 5-mL BHI medium and grown until the stationary phase in an anaerobic chamber (37°C, 250 RPM). It was then diluted in the same medium to obtain a final inoculum of 10^6^ cells/mL, followed by treatment with CsOSM (5 mg/mL). We performed biological triplicates of this experiment, sampled at 0, 3, 9, 18, 24, 48, 72, 96, 120, 144, and 168 hours.

For the dose response assay of DCA, 10^6^ cells/mL of *C. difficile* was cultured in BHI medium containing nine different concentrations of DCA (0.5, 10, 20, 40, 80, 160, 310, 630, and 1,250 µM) (Sigma-Aldrich, Catalog # D2510). The mono-cultures were incubated for a 168-hour time course, and growth was determined at indicated timepoints by measuring optical density (OD) at 595 nm.

The pH of the cultures with CsOSM at different concentrations was recorded using a pH meter (VWR phenomenal PH 1100L, Germany).

### Cell counting

The *C. difficile* cell number was measured using the Quantom Tx microbial cell counter (Logos Biosystem, South Korea) and QUANTOM Total Cell Staining Kit according to the manufacturer’s instructions. The bacterial pellets were resuspended in sterile SF, and the samples were diluted 10 times. Ten microliters of diluted samples was mixed well with 1 mL of Quantom Total Cell Staining Dye, 1 mL of Quantom Total Cell Staining Enhancer, and 8 mL of Quantom Cell Loading Buffer I. From the resulting mixture, 6 µL was loaded on a Quantom M50 Cell Counting Slide and centrifuged at 300 × *g* for 10 minutes in a Quantom Centrifuge. Then, the samples were counted with the Quantom Tx Microbial Cell Counter with the following parameters: light intensity, level 5, size gating, ~0.3 to 50 µm; roundness, 25%; declustering level, 10; and detection sensitivity, 9.

### ELISA

The samples collected from *C. difficile* cultured with CsOSM (5 mg/mL) in culture flasks were used for toxin assays. The production of TcdA and TcdB was determined using a commercially available ELISA kit (tgcBIOMICS, Rhein, Germany) as recommended by the manufacturer. As a positive control, a *C. difficile* toxin A/B mix provided by the manufacturer was used.

### Proteomics sample preparation

In order to precipitate the proteins present in the culture supernatants, 800 µL of pre-cooled 100% acetone was added to 200 µL of culture supernatants, vortexed, and incubated at −20°C for 60 minutes. Then, the samples were centrifuged at 15,000 × *g* for 10 minutes at 4°C to obtain the precipitated proteins as a pellet. The supernatants were then decanted carefully without dislodging the protein pellets, which were allowed to dry at room temperature for 30 minutes. The dried pellets were then resuspended in a buffer (100 µL) containing 5% SDS (UltraPure, Thermo Fisher Scientific, USA), 50 mM TEAB (Thermo Fisher Scientific, USA), pH 7.55, and boiled for 15 minutes.

From the resuspended mix, proteins were purified and digested by trypsin and Lys-C using the protein aggregation capture PAC protocol (67). Digested peptides were acidified to 1% TFA and desalted on SDB-RPS StageTips (68). Peptides were separated on home-packed 50-cm, 75-µM ID columns packed with ReproSil-Pur C18-AQ beads (1.9 µm) (Dr. Maisch) on an EASY-nLC 1200 ultra-high-pressure system and injected via a CaptiveSpray source and a 10-μm emitter into a timsTOFpro mass spectrometer (Bruker). Peptides were loaded in buffer A (0.1% formic acid) and separated applying a non-linear gradient of 5–60% buffer B (0.1% formic acid, 80% acetonitrile) at a flow rate of 300 nL/minute over 100 minutes, and MS data were acquired in the PASEF mode (69).

### Proteomics data analysis

Raw mass spectrometry data were analyzed with MaxQuant (v1.6.15.0). Peak lists were searched against a combined Uniprot FASTA database consisting of proteome ID UP000001978, as well as 262 common contaminants by the integrated Andromeda search engine. The false discovery rate was 1% for both peptides (minimum length of 7 amino acids) and proteins. “Match between runs” was enabled with a Match time window of 0.7 and a Match ion mobility window of 0.05 minutes. The MaxLFQ algorithm determined relative protein amounts with a minimum ratio count of two.

### Transmission electron microscopy

Pellets of bacteria were fixed with 2% (vol/vol) glutaraldehyde in 0.05 M sodium phosphate buffer (pH 7.2). The pellets were embedded in agarose, rinsed three times in 0.15 M sodium phosphate buffer (pH 7.2), and subsequently postfixed in 1% (wt/vol) OsO_4_ with 0.05 M K_3_Fe(CN)_6_ in 0.12 M sodium phosphate buffer (pH 7.2) for 2 hours. The specimens were dehydrated in a graded series of ethanol, transferred to propylene oxide, and embedded in Epon according to standard procedures. Sections, approximately 60-nm thick, were cut with a Ultracut 7 (Leica, Wienna, Austria) and collected on copper grids with Formvar supporting membranes, stained with uranyl acetate and lead citrate, and subsequently examined with a Philips CM 100 Transmission EM (Philips, Eindhoven, The Netherlands), operated at an accelerating voltage of 80 kV. Digital images were recorded with an OSIS Veleta digital slow scan 2k × 2k CCD camera and the ITEM software package.

### TEM image cell counting

We performed manual cell counting on TEM images of *C. difficile* mono-culture and *C. difficile* mono-culture treated with CsOSM. We counted three types of cell morphology: normal vegetative cells, elongated vegetative cells, and spores (Fig. S7). We did not count dead cells—as they were only fragments of cells, it was not possible to count them reliably. Table S7 corresponds to the count of total cells and three types of cell morphology in the images at 10-µm resolution (Fig. S7B).

### Visualization

All the visualization outputs were generated using the ggplot2 (v3.3.5 [70]) R package. To generate a clear trend in some of the visualizations, a *lo*cal regr*ess*ion (LOESS) fit was used, implemented in the R function lm. In addition, principal component analysis was performed using the log-transformed gene expression profiles and prcomp from the stats (v4.0.3) (71) R package with center and scale parameters (center = T, sca = T).

### Statistical analysis

A student’s *t*-test was performed to assess any significant differences between *C. difficile* mono-culture and *C. difficile* batches treated with freeze-dried CsOSM; control and treatment samples; or BHI and CsOSM media. *P* values were corrected for multiple testing using FDR correction.

## Data Availability

Sequencing data have been deposited in the NCBI short read archive under the BioProject identifier PRJNA985904.

## SUPPLEMENTAL MATERIAL

Supplemental Figures spectrum.03933-22-s0001.docx Figures S1 to S7.

Supplemental Tables spectrum.03933-22-s0002.docx Tables S1 to S7.

ASM does not own the copyrights to Supplemental Material that may be linked to, or accessed through, an article. The authors have granted ASM a non-exclusive, world-wide license to publish the Supplemental Material files. Please contact the corresponding author directly for reuse.
