# Supplementary material for: Clostridium scindens secretome suppresses virulence gene expression of Clostridioides difficile in a bile acid-independent manner
Source: Microbiol Spectr. 2023 Sep 26;11(5):e03933-22. doi: 10.1128/spectrum.03933-22 (PMC10581174; doi:10.1128/spectrum.03933-22)
Supplement: Supplemental Figures — Figures S1 to S7. [file spectrum.03933-22-s0001.docx]

**SUPPLEMENTARY FIGURES**


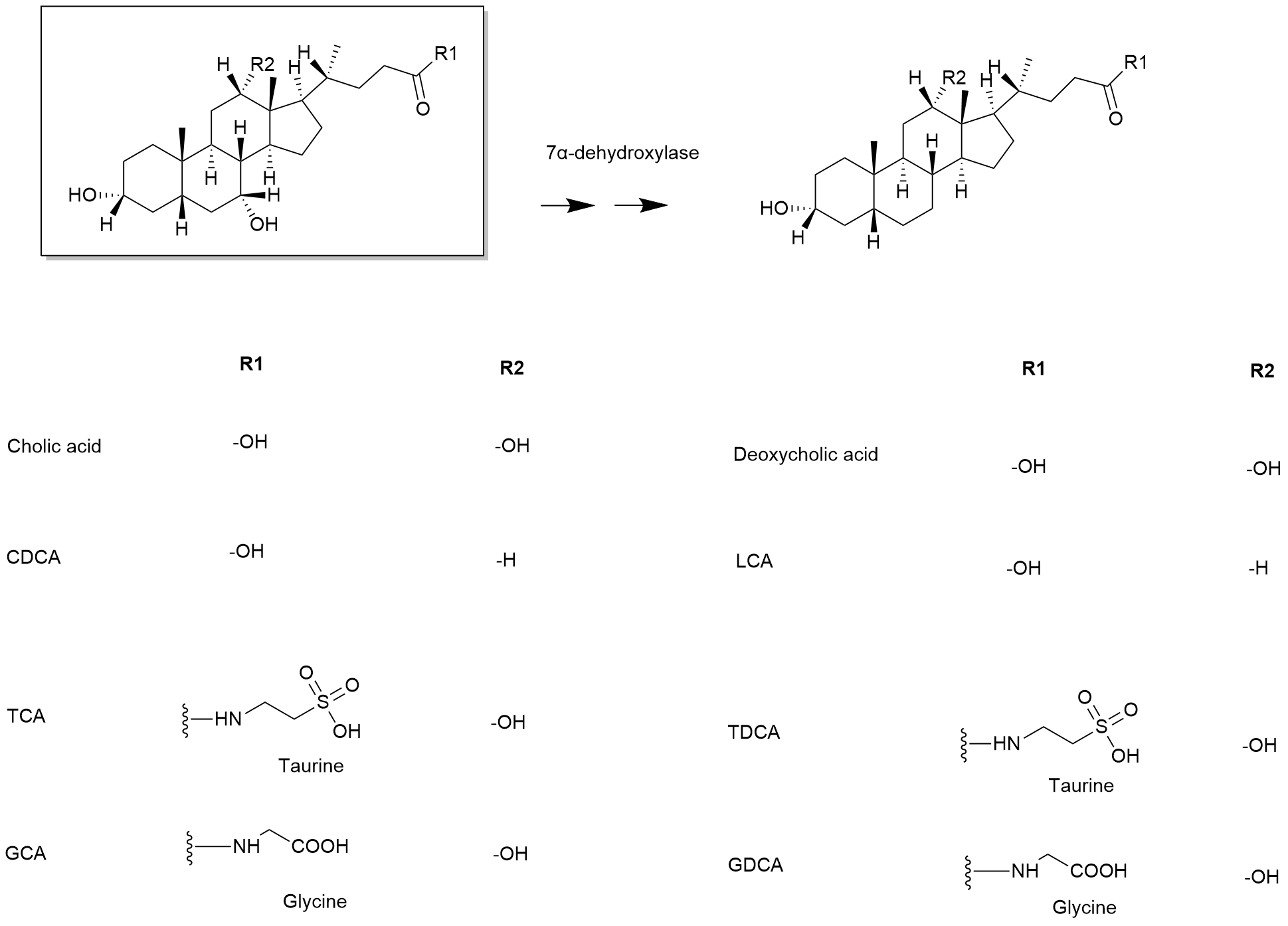


**Figure S1.** Biotransformation of primary BAs CA, TCD and GCA into secondary BAs DCA, TDCA and GDCA, and related enzymes.

**
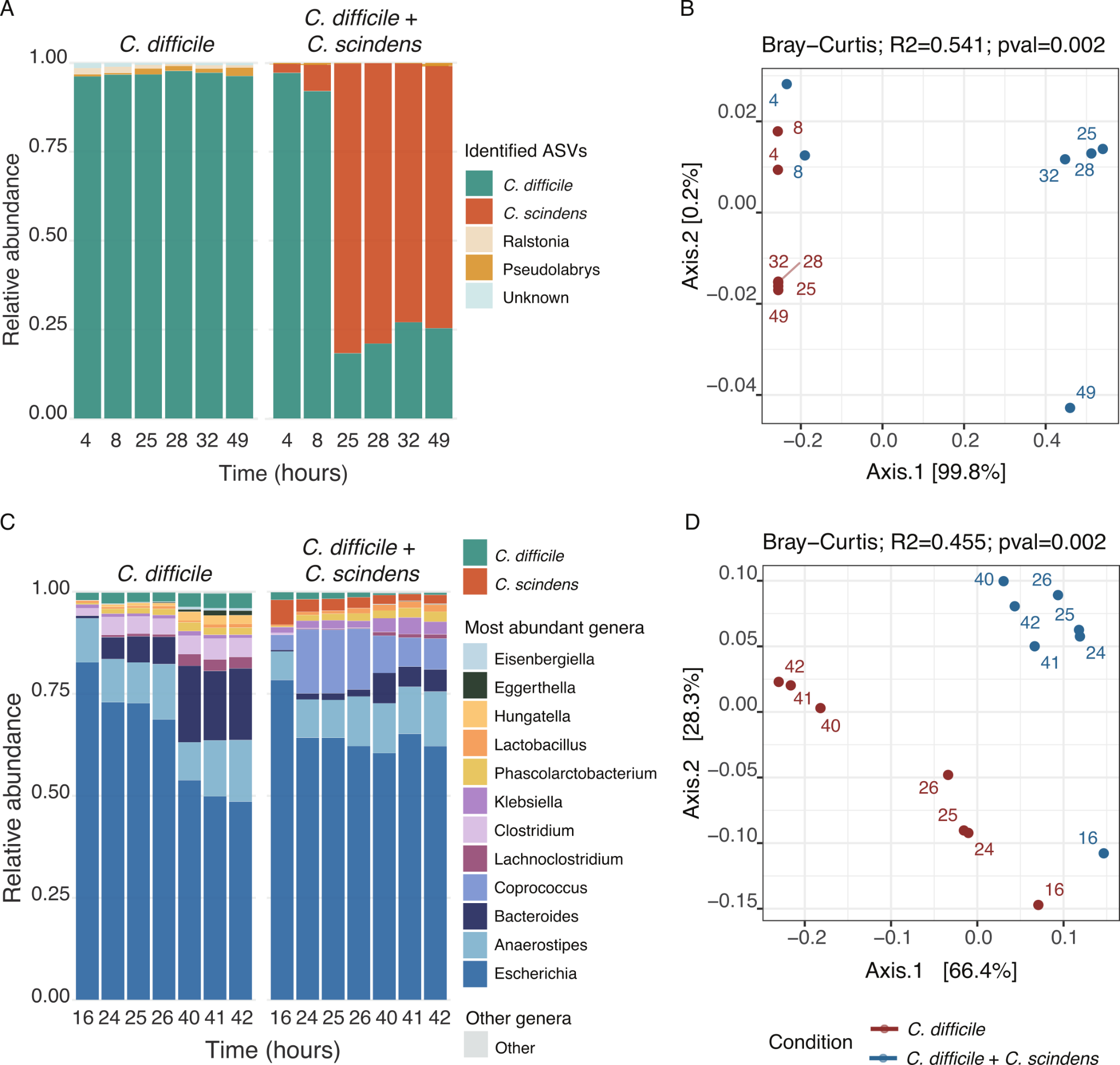
**

**Figure S2.** Taxonomic profiles. **A)** Relative abundance for the five ASVs identified across the samples in the continuous culture setup using the SILVA database [(55)](https://paperpile.com/c/pUqJy1/QDwpS) and DADA2 [(54)](https://paperpile.com/c/pUqJy1/mY3vp). **B)** Projection of the first two principal coordinates based on Bray–Curtis dissimilarity between the microbiome composition in the control (red) and treatment (blue) conditions of the continuous culture setup. **C)** Relative abundance for the 15 most abundant genera across the samples in the batch culture setup using MetaPhlAn3 [(63)](https://paperpile.com/c/pUqJy1/rf6nX). **(D)** Projection of the first two principal coordinates based on Bray–Curtis dissimilarity between the microbiome composition in the control and treatment conditions of the batch culture setup.


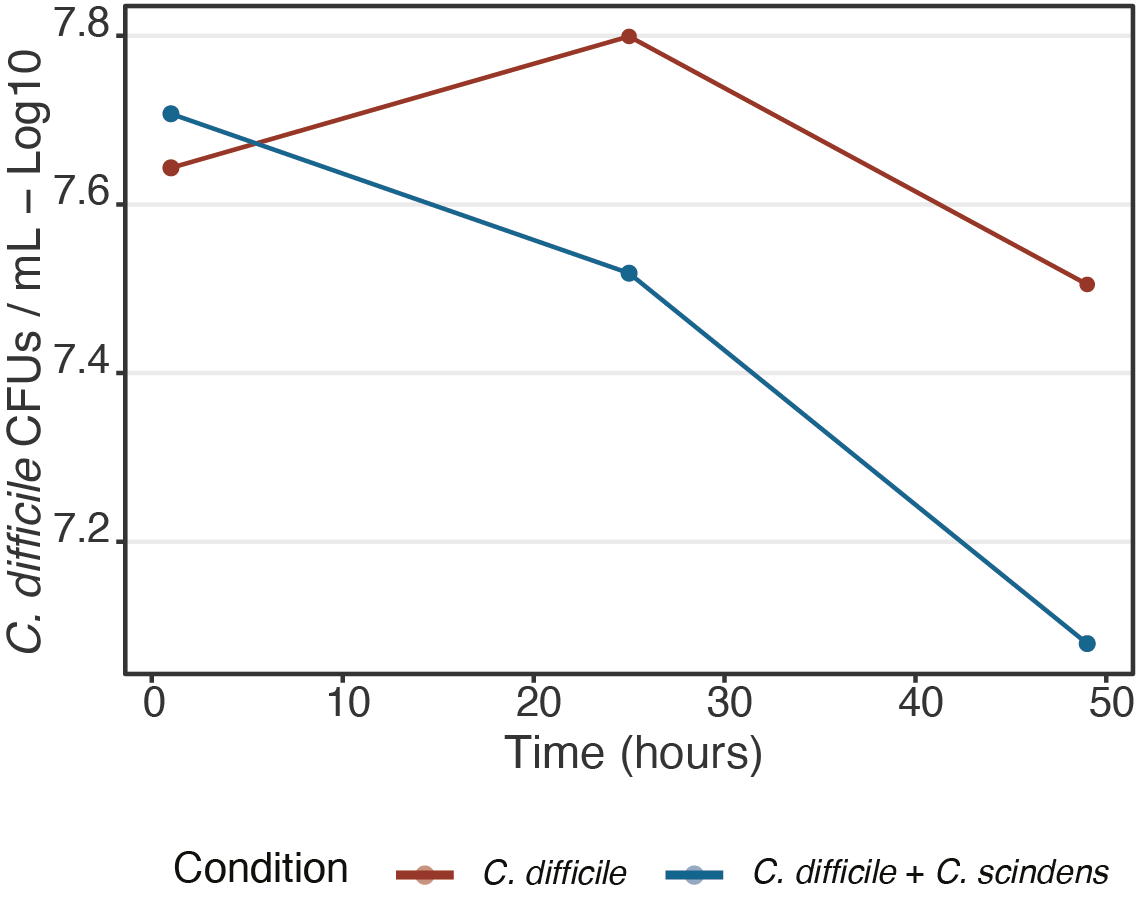


**Figure S3.** Quantification of *C. difficile* colony forming units (CFU).


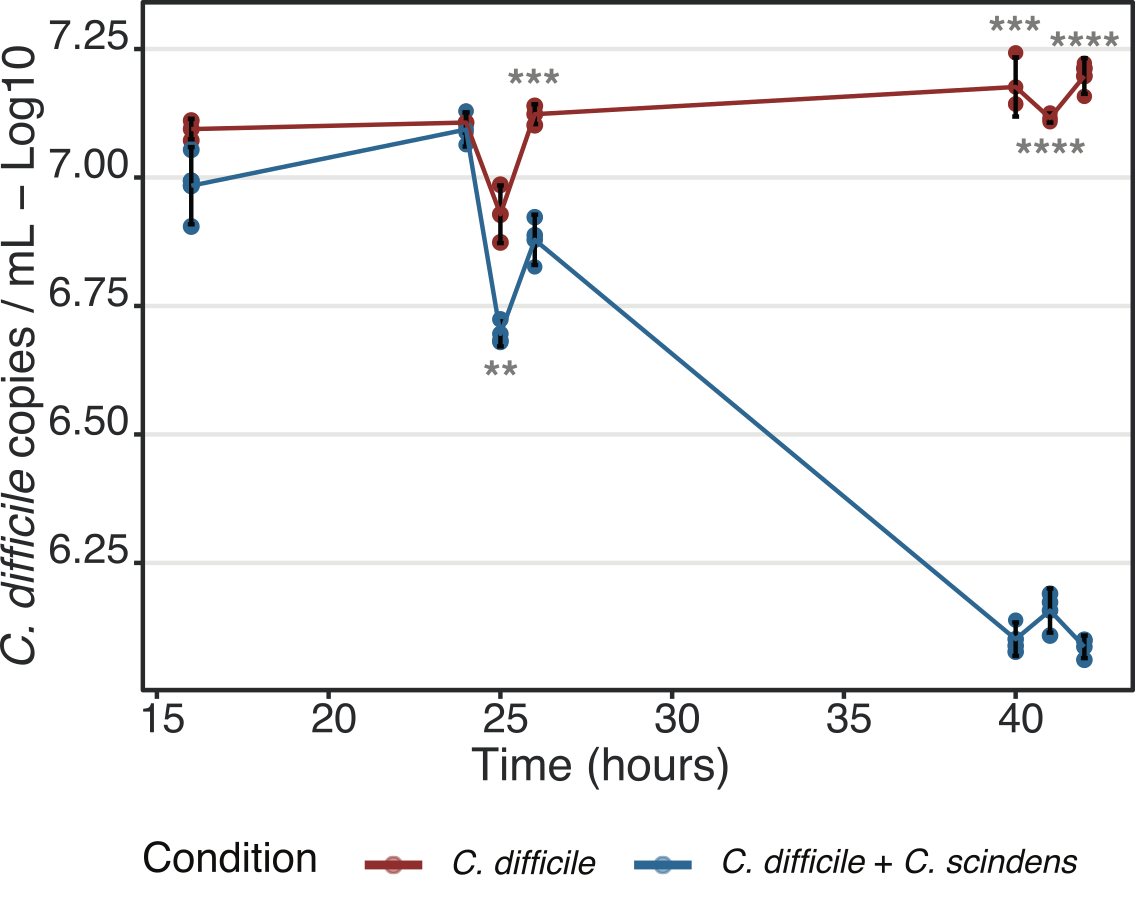


**Figure S4.** Quantification of *C. difficile* total number of cells via qPCR detection. ** adjusted P < 0.01, *** adjusted P < 0.0001 and **** adjusted P < 0.00001, Student’s t-test. n=3 independent experiments.


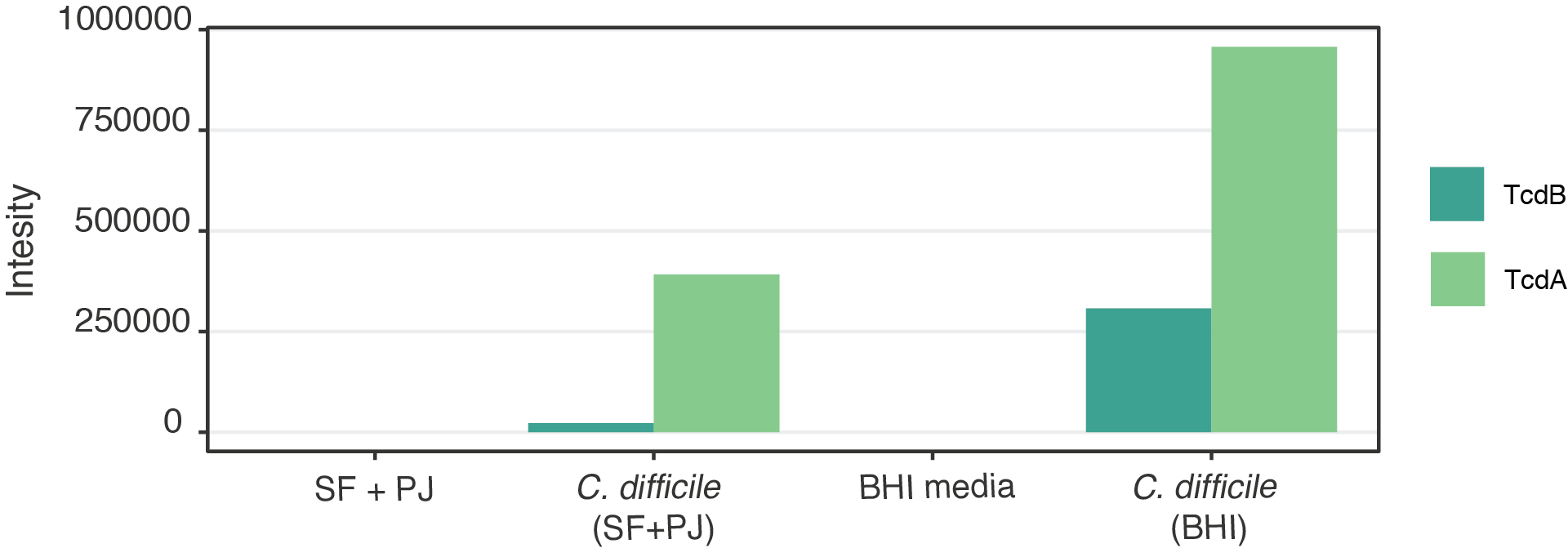


**Figure S5.** Proteomics-based quantification of TcdA and TcdB toxin abundance in *C. difficile* cultured in two different media using culture flasks: (i) Standard nutritional medium feed (SF) with pancreatic/bile juice (PJ) and (ii) Brain heart infusion (BHI) medium. Matching control media without *C. difficile* cells are also shown.

**
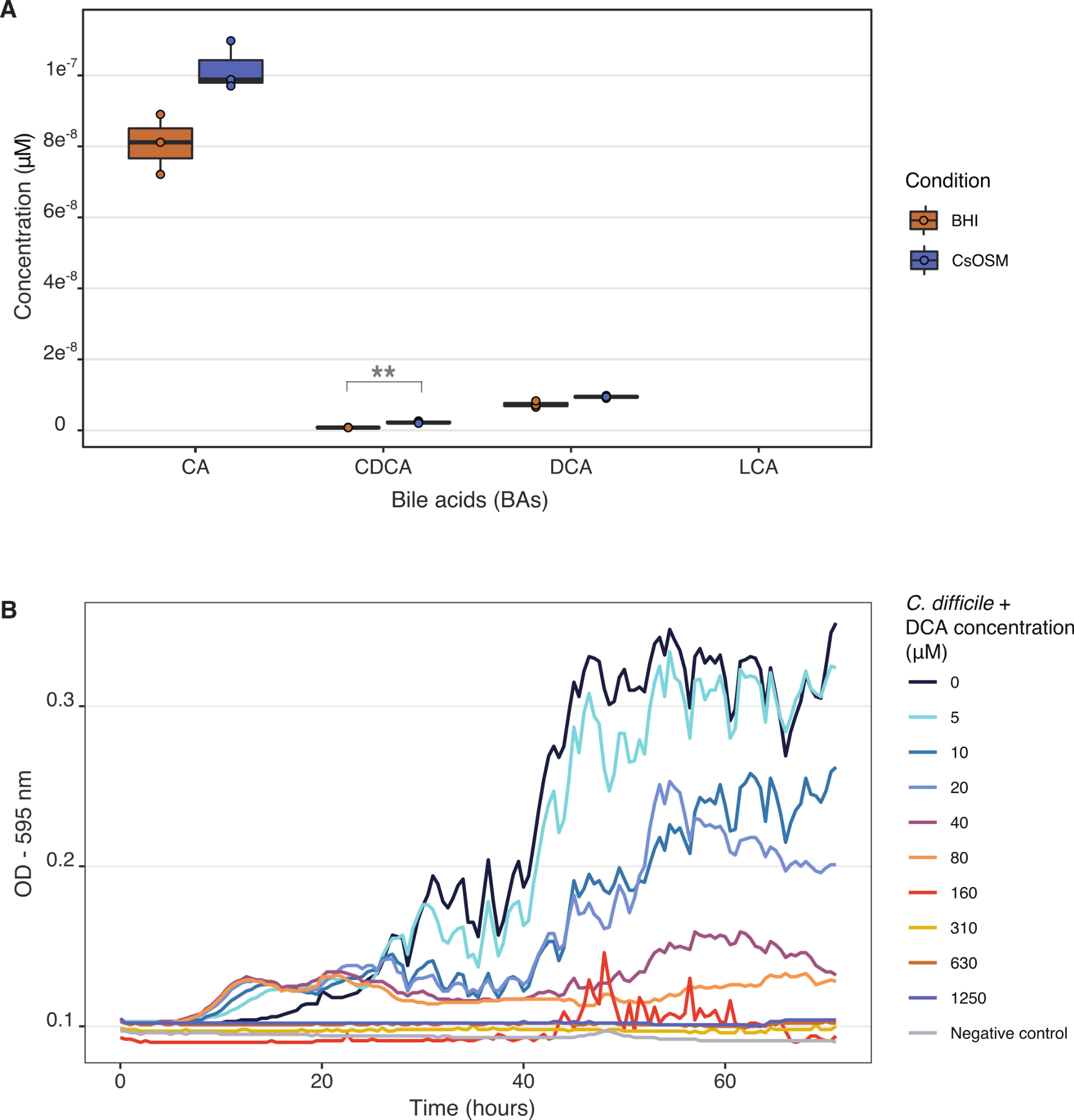
**

**Figure S6** **A)** Quantification of bile acids: CA, CDCA, DCA and LCA in Brain heart infusion (BHI) and CsOSM, using targeted metabolomics. ** adjusted P < 0.01, Student’s t-test. n=3 independent experiments.. **B)** Optical density (OD) at 595 nm of *C. difficile* mono-cultures in a medium with the supplement of different concentrations of secondary bile acid DCA for 71 hours.


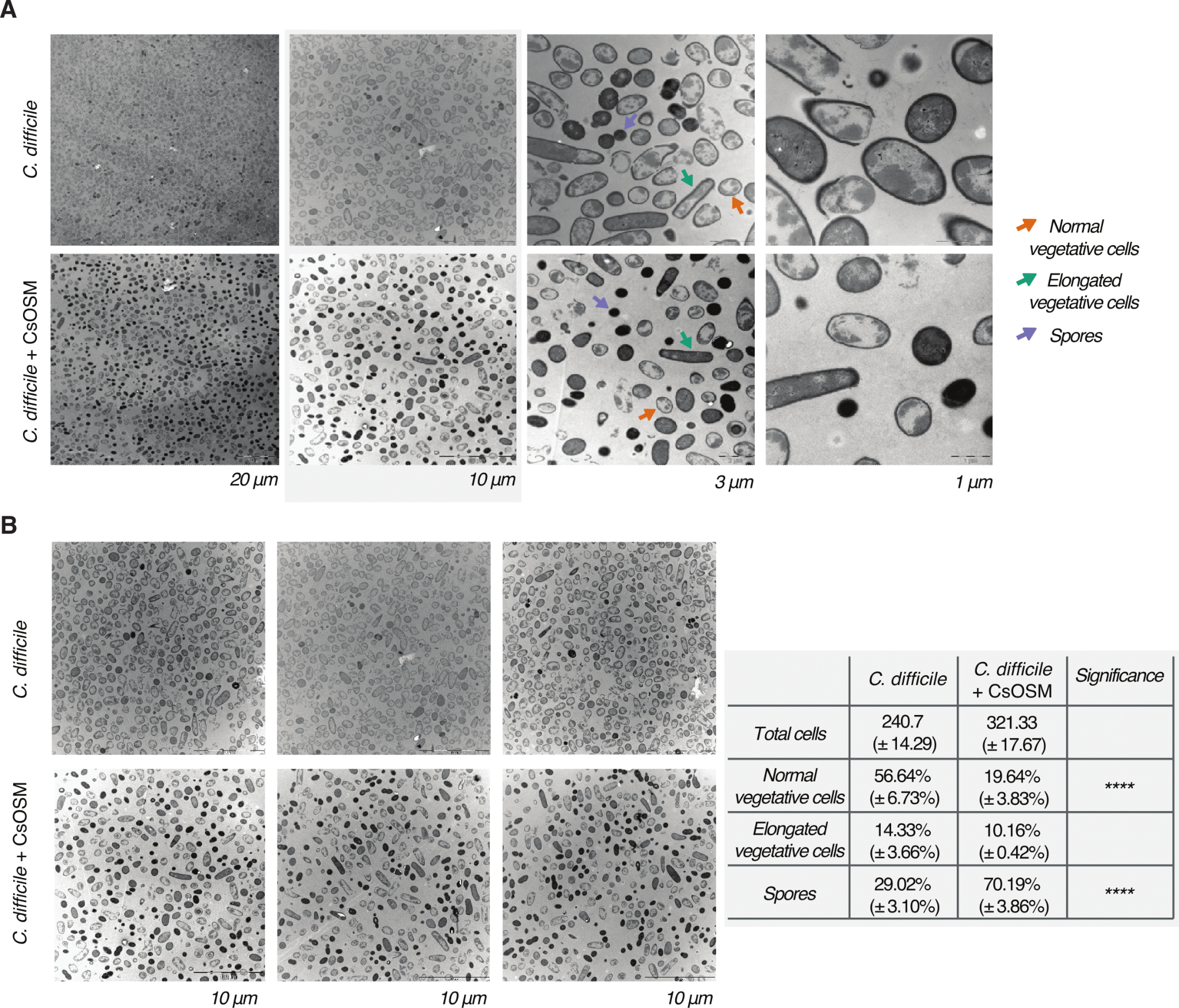


**Figure S7.** TEM images of *C. difficile* mono-culture and *C. difficile* mono-culture treated with CsOSM. **A)** Scales represent 20 μm, 10 μm, 3 μm and 1 μm. **B)** Counting three types of cell morphology at 10 μm resolution: normal vegetative cells, elongated vegetative cells, and spores. We did not count dead cells - as they were only fragments of cells, it was not possible to count them reliably. The table corresponds to total cells and the percentage (mean and standard deviation) of the three types of morphology cells in the images (n=3, Student’s t-test, **** adjusted P < 0.0001).
