# Supplementary material for: Clostridium scindens secretome suppresses virulence gene expression of Clostridioides difficile in a bile acid-independent manner
Source: Microbiol Spectr. 2023 Sep 26;11(5):e03933-22. doi: 10.1128/spectrum.03933-22 (PMC10581174; doi:10.1128/spectrum.03933-22)
Supplement: Supplemental Tables — Tables S1 to S7. [file spectrum.03933-22-s0002.docx]

**SUPPLEMENTARY TABLES**

| **Condition** | ***C. difficile*** | | | | | | |
| --- | --- | --- | --- | --- | --- | --- | --- |
| **time (hours)** | **1** | **4** | **8** | **25** | **28** | **32** | **49** |
| **CA** | 2609.60 | 2005.67 | 288.23 | 1608.21 | 1424.51 | 1599.82 | 455.46 |
| **CDCA** | 181.51 | 0.00 | 3.09 | 129.45 | 99.85 | 72.02 | 23.65 |
| **DCA** | 1011.37 | 525.48 | 38.64 | 698.61 | 447.51 | 526.30 | 132.42 |
| **GCA** | 11939.21 | 12695.19 | 2220.36 | 7801.35 | 7646.57 | 7984.60 | 3035.72 |
| **GCDCA** | 634.12 | 615.62 | 91.19 | 410.70 | 424.37 | 410.01 | 112.74 |
| **GDCA** | 2084.54 | 2495.61 | 360.98 | 1616.89 | 1736.90 | 1972.42 | 538.99 |
| **GLCA** | 0.00 | 0.00 | 0.00 | 0.00 | 0.00 | 0.00 | 0.00 |
| **GUDCA** | 0.00 | 0.00 | 0.00 | 0.00 | 0.00 | 0.00 | 0.00 |
| **HDCA** | 0.00 | 0.00 | 0.00 | 0.00 | 0.00 | 0.00 | 0.00 |
| **LCA** | 0.00 | 0.00 | 0.00 | 0.00 | 0.00 | 0.00 | 0.00 |
| **MCA(a)** | 0.00 | 0.00 | 0.00 | 0.00 | 0.00 | 0.00 | 0.00 |
| **MCA(b)** | 0.00 | 0.00 | 0.00 | 0.00 | 0.00 | 0.00 | 0.00 |
| **MCA(g)** | 0.00 | 0.00 | 0.00 | 0.00 | 0.00 | 0.00 | 0.00 |
| **MCA(o)** | 0.00 | 0.00 | 0.00 | 0.00 | 0.00 | 0.00 | 0.00 |
| **Murocholic Acid** | 0.00 | 0.00 | 0.00 | 0.00 | 0.00 | 0.00 | 0.00 |
| **TCA** | 27105.46 | 30871.72 | 4616.87 | 19214.64 | 18730.26 | 16671.76 | 6664.48 |
| **TCDCA** | 702.32 | 1053.31 | 160.34 | 687.94 | 547.97 | 640.71 | 189.62 |
| **TDCA** | 2706.04 | 3559.53 | 689.40 | 2366.37 | 2709.15 | 2971.04 | 849.01 |
| **THDCA** | 0.00 | 0.00 | 0.00 | 0.00 | 0.00 | 0.00 | 0.00 |
| **TLCA** | 43.79 | 0.00 | 0.00 | 35.19 | 0.00 | 0.00 | 0.00 |
| **TMCA(a)** | 0.00 | 0.00 | 0.00 | 0.00 | 0.00 | 0.00 | 0.00 |
| **TMCA(b)** | 0.00 | 0.00 | 0.00 | 0.00 | 0.00 | 0.00 | 0.00 |
| **TMCA(g)** | 0.00 | 0.00 | 0.00 | 0.00 | 0.00 | 0.00 | 0.00 |
| **TMCA(o)** | 0.00 | 0.00 | 0.00 | 0.00 | 0.00 | 0.00 | 0.00 |
| **TUDCA** | 0.00 | 0.00 | 0.00 | 0.00 | 0.00 | 0.00 | 0.00 |
| **UDCA** | 0.00 | 0.00 | 0.00 | 0.00 | 0.00 | 0.00 | 0.00 |

| **Condition** | ***C. difficile* + *C. scindens*** | | | | | | |
| --- | --- | --- | --- | --- | --- | --- | --- |
| **time (hours)** | **1** | **4** | **8** | **25** | **28** | **32** | **49** |
| **CA** | 2344.26 | 342.56 | 7819.96 | 5712.28 | 744.86 | 98.21 | 104.18 |
| **CDCA** | 124.88 | 16.41 | 571.78 | 0.00 | 0.00 | 0.00 | 0.00 |
| **DCA** | 922.00 | 92.93 | 3585.12 | 9876.39 | 4022.16 | 889.59 | 788.63 |
| **GCA** | 11336.47 | 1296.47 | 15641.91 | 789.12 | 0.00 | 0.00 | 20.57 |
| **GCDCA** | 611.00 | 45.85 | 797.68 | 54.83 | 1.67 | 0.00 | 0.00 |
| **GDCA** | 2382.97 | 264.93 | 2988.92 | 358.71 | 26.64 | 0.00 | 5.58 |
| **GLCA** | 0.00 | 0.00 | 0.00 | 0.00 | 0.00 | 0.00 | 0.00 |
| **GUDCA** | 0.00 | 0.00 | 0.00 | 0.00 | 0.00 | 0.00 | 0.00 |
| **HDCA** | 0.00 | 0.00 | 0.00 | 0.00 | 0.00 | 0.00 | 0.00 |
| **LCA** | 0.00 | 0.00 | 0.00 | 395.52 | 188.36 | 0.00 | 0.00 |
| **MCA(a)** | 0.00 | 0.00 | 0.00 | 0.00 | 0.00 | 0.00 | 0.00 |
| **MCA(b)** | 0.00 | 0.00 | 0.00 | 0.00 | 0.00 | 0.00 | 0.00 |
| **MCA(g)** | 0.00 | 0.00 | 0.00 | 0.00 | 0.00 | 0.00 | 0.00 |
| **MCA(o)** | 0.00 | 0.00 | 0.00 | 0.00 | 0.00 | 0.00 | 0.00 |
| **Murocholic Acid** | 0.00 | 0.00 | 0.00 | 0.00 | 0.00 | 0.00 | 0.00 |
| **TCA** | 29356.07 | 3086.84 | 42709.17 | 42034.27 | 11061.16 | 2368.99 | 1622.66 |
| **TCDCA** | 1412.37 | 98.23 | 1549.74 | 1298.93 | 346.24 | 78.13 | 54.63 |
| **TDCA** | 3316.95 | 434.04 | 5475.81 | 4606.71 | 1414.70 | 380.58 | 227.74 |
| **THDCA** | 0.00 | 0.00 | 0.00 | 0.00 | 0.00 | 0.00 | 0.00 |
| **TLCA** | 0.00 | 0.00 | 0.00 | 71.50 | 29.12 | 2.86 | 4.14 |
| **TMCA(a)** | 0.00 | 0.00 | 0.00 | 0.00 | 0.00 | 0.00 | 0.00 |
| **TMCA(b)** | 0.00 | 0.00 | 0.00 | 0.00 | 0.00 | 0.00 | 0.00 |
| **TMCA(g)** | 0.00 | 0.00 | 0.00 | 0.00 | 0.00 | 0.00 | 0.00 |
| **TMCA(o)** | 0.00 | 0.00 | 0.00 | 0.00 | 0.00 | 0.00 | 0.00 |
| **TUDCA** | 0.00 | 0.00 | 0.00 | 0.00 | 0.00 | 0.00 | 0.00 |
| **UDCA** | 0.00 | 0.00 | 0.00 | 0.00 | 0.00 | 0.00 | 0.00 |

**Table S1.** BAs intensities quantified (ng/L) in control (*C. difficile*) and treatment (*C. difficile + C. scindens*) conditions using a continuous culture setup.

| **CsOSM (mg/mL)** | **R1** | **R2** | **R3** | **Average pH value with standard deviation** |
| --- | --- | --- | --- | --- |
| **CK** | 6.7 | 6.7 | 6.7 | 6.7 ± 0 |
| **1.25** | 6.8 | 6.7 | 6.7 | 6.7 ± 0.05 |
| **2.5** | 6.7 | 6.8 | 6.6 | 6.7 ± 0.1 |
| **5** | 6.7 | 6.7 | 6.6 | 6.6 ± 0.05 |
| **10** | 6.7 | 6.7 | 6.7 | 6.7 ± 0.1 |
| **20** | 6.8 | 6.7 | 6.6 | 6.7 ± 0.1 |
| **40** | 6.7 | 6.7 | 6.6 | 6.6 ± 0.05 |
| **80** | 6.7 | 6.8 | 6.7 | 6.7 ± 0.05 |

**Table S2.** pH measurement records of *C. difficile* culture when different amount of CsOSM were added.

| **Media** | **BHI** | | | **CsOSM** | | |
| --- | --- | --- | --- | --- | --- | --- |
| **Sample replicates** | **1** | **2** | **3** | **1** | **2** | **3** |
| **CA** | 2.95E-02 | 3.32E-02 | 3.64E-02 | 4.48E-02 | 4.04E-02 | 3.96E-02 |
| **CDCA** | 3.35E-04 | 3.01E-04 | 3.02E-04 | 1.04E-03 | 8.56E-04 | 7.84E-04 |
| **DCA** | 2.57E-03 | 2.80E-03 | 3.26E-03 | 3.85E-03 | 3.55E-03 | 3.71E-03 |
| **GCA** | 9.16E-03 | 1.06E-02 | 1.24E-02 | 1.34E-04 | 1.15E-04 | 3.24E-05 |
| **GCDCA** | 4.36E-04 | 5.10E-04 | 6.96E-04 | 1.43E-06 | 6.67E-06 | 0.00 |
| **GDCA** | 1.91E-03 | 2.15E-03 | 2.54E-03 | 4.71E-05 | 4.00E-05 | 0.00 |
| **GLCA** | 0.00 | 0.00 | 0.00 | 0.00 | 0.00 | 0.00 |
| **GUDCA** | 0.00 | 8.03E-06 | 0.00 | 0.00 | 0.00 | 0.00 |
| **HDCA** | 0.00 | 0.00 | 0.00 | 0.00 | 0.00 | 0.00 |
| **LCA** | 0.00 | 0.00 | 0.00 | 0.00 | 0.00 | 0.00 |
| **MCA(a)** | 0.00 | 0.00 | 0.00 | 0.00 | 0.00 | 0.00 |
| **MCA(b)** | 0.00 | 0.00 | 0.00 | 0.00 | 0.00 | 0.00 |
| **MCA(g)** | 0.00 | 0.00 | 0.00 | 0.00 | 0.00 | 0.00 |
| **MCA(o)** | 0.00 | 0.00 | 0.00 | 0.00 | 0.00 | 0.00 |
| **Murocholic Acid** | 0.00 | 5.98E-05 | 1.14E-05 | 0.00 | 0.00 | 0.00 |
| **TCA** | 4.24E-03 | 5.03E-03 | 7.45E-03 | 6.32E-04 | 6.08E-04 | 1.71E-04 |
| **TCDCA** | 2.64E-04 | 2.60E-04 | 4.03E-04 | 3.40E-05 | 3.34E-05 | 0.00 |
| **TDCA** | 4.12E-04 | 4.34E-04 | 7.43E-04 | 4.23E-05 | 1.04E-05 | 0.00 |
| **THDCA** | 0.00 | 0.00 | 0.00 | 0.00 | 0.00 | 0.00 |
| **TLCA** | 0.00 | 0.00 | 0.00 | 0.00 | 0.00 | 0.00 |
| **TMCA(a)** | 0.00 | 0.00 | 0.00 | 0.00 | 0.00 | 0.00 |
| **TMCA(b)** | 0.00 | 0.00 | 0.00 | 0.00 | 0.00 | 0.00 |
| **TMCA(g)** | 0.00 | 0.00 | 0.00 | 0.00 | 0.00 | 0.00 |
| **TMCA(o)** | 0.00 | 0.00 | 0.00 | 0.00 | 0.00 | 0.00 |
| **TUDCA** | 5.27E-05 | 4.69E-05 | 6.20E-05 | 4.07E-05 | 3.70E-05 | 3.24E-05 |
| **UDCA** | 2.50E-03 | 2.89E-03 | 2.68E-03 | 2.33E-03 | 2.03E-03 | 1.86E-03 |

**Table S3.** BAs intensities quantified (ng/L) in Brain heart infusion (BHI) and CsOSM (n=3).

| **Time (min)** | **%B** |
| --- | --- |
| 0 | 20 |
| 20 | 55 |
| 26 | 100 |
| 28.1 | 100 |
| 28.2 | 20 |
| 30 | 20 |

**Table S4.** Gradients used in LC-MS analysis for the targeted quantification of BAs.

| **Compound** | **Transition** | **Dwell Time (s)** | **Cone Voltage (V)** | **Collision Energy** |
| --- | --- | --- | --- | --- |
| **LCA** | 375.47 > 375.47 | 0.011 | 4 | 14 |
| **LCA-d5** | 380.53 > 380.53 | 0.011 | 12 | 28 |
| **DCA** | 391.49 > 345.43 | 0.011 | 52 | 32 |
| **UDCA and Isomers** | 391.53 > 391.53 | 0.011 | 4 | 16 |
| **UDCA-d4** | 395.49 > 395.49 | 0.011 | 4 | 18 |
| **DCA-d4** | 395.54 > 349.48 | 0.011 | 68 | 32 |
| **CA and isomers** | 407.50 > 407.50 | 0.011 | 2 | 20 |
| **CA-d4** | 411.55 > 411.55 | 0.011 | 2 | 14 |
| **GLCA** | 432.48 > 74.08 | 0.011 | 34 | 32 |
| **GDCA and isomers** | 448.53 > 74.08 | 0.011 | 62 | 34 |
| **GUDCA-d4** | 452.56 > 74.08 | 0.011 | 60 | 34 |
| **GCA** | 464.55 > 74.07 | 0.011 | 14 | 34 |
| **GCA-d4** | 468.55 > 74.08 | 0.011 | 62 | 38 |
| **TLCA** | 482.53 > 80.06 | 0.011 | 98 | 60 |
| **TLCA-d4** | 486.59 > 80.06 | 0.011 | 72 | 62 |
| **TDCA and isomers** | 498.56 > 80.06 | 0.011 | 86 | 62 |
| **TDCA-d4** | 502.54 > 80.00 | 0.011 | 96 | 64 |
| **TCA and isomers** | 514.52 > 80.07 | 0.011 | 80 | 64 |
| **TCA-d4** | 518.60 > 80.06 | 0.011 | 98 | 58 |

**Table S5.** Bile acid MRM transitions and conditions.

| **Bile Acid** | **RT (min)** | **Internal Standard** |
| --- | --- | --- |
| **CA** | 17.17 | CA-d4 |
| **CA-d4** | 17.16 | N/A |
| **CDCA** | 19.74 | CDCA-d4 |
| **CDCA-d4** | 19.73 | N/A |
| **DCA** | 20.04 | DCA-d4 |
| **DCA-d4** | 20.04 | N/A |
| **GCA** | 14.84 | GCA-d4 |
| **GCA-d4** | 14.83 | N/A |
| **GCDCA** | 17.56 | GCDCA-d4 |
| **GCDCA-d4** | 17.55 | N/A |
| **GDCA** | 18.02 | GDCA-d4 |
| **GDCA-d4** | 17.99 | N/A |
| **GLCA** | 20.39 | TCDCA-d4 |
| **GUDCA** | 14.47 | GUDCA-d4 |
| **GUDCA-d4** | 14.43 | N/A |
| **HDCA** | 17.53 | UDCA-d4 |
| **LCA** | 22.3 | LCA-d5 |
| **LCA-d5** | 22.26 | N/A |
| **MCA alpha** | 14.78 | CA-d4 |
| **MCA beta** | 15.18 | CA-d4 |
| **MCA gamma** | 16.35 | CA-d4 |
| **MCA omega** | 14.4 | CA-d4 |
| **Murocholic acid** | 16.4 | UDCA-d4 |
| **TCA** | 14.03 | TCA-d4 |
| **TCA-d4** | 14.02 | N/A |
| **TCDCA** | 16.9 | TCDCA-d4 |
| **TCDCA-d4** | 16.87 | N/A |
| **TDCA** | 17.47 | TCDCA-d4 |
| **THDCA** | 13.51 | TCDCA-d4 |
| **TLCA** | 20 | TLCA-d4 |
| **TLCA-d4** | 19.98 | N/A |
| **TMCA alpha** | 8.26 | TCA-d4 |
| **TMCA beta** | 8.59 | TCA-d4 |
| **TMCA gamma** | 11.76 | TCA-d4 |
| **TMCA omega** | 17.65 | TCA-d4 |
| **TUDCA** | 13.31 | TCDCA-d4 |
| **UDCA** | 17.31 | UDCA-d4 |
| **UDCA-d4** | 17.28 | N/A |

**Table S6.** BAs retention time and internal standard used for quantitation. RT, retention time; N/A, not applicable.

| **Condition** | **Image replicate** | **Vegetative cells** | **Elongated vegetative cells** | **Spores** | **Total cells** |
| --- | --- | --- | --- | --- | --- |
| *C. difficile* | 1 | 156 | 25 | 63 | 244 |
|  | 2 | 114 | 39 | 72 | 225 |
|  | 3 | 140 | 39 | 74 | 253 |
| *C. difficile*  + CsOSM | 1 | 72 | 31 | 198 | 301 |
|  | 2 | 55 | 35 | 243 | 333 |
|  | 3 | 61 | 32 | 237 | 330 |

**Table S7.** Count of total cells and three types of cell morphology in the images at 10 μm resolution (**Figure S7B**). We did not count dead cells - as they were only fragments of cells, it was not possible to count them reliably.
